# Uridine-sensitized screening identifies demethoxy-coenzyme Q and NUDT5 as regulators of nucleotide synthesis

**DOI:** 10.1038/s42255-025-01419-2

**Published:** 2025-11-13

**Authors:** Abigail Strefeler, Zakery N. Baker, Sylvain Chollet, Mads M. Foged, Rachel M. Guerra, Julijana Ivanisevic, Hector Gallart-Ayala, David J. Pagliarini, Alexis A. Jourdain

**Affiliations:** 1 Department of Immunobiology, University of Lausanne, Epalinges, Switzerland; 2 Department of Cell Biology & Physiology, Washington University School of Medicine, St. Louis, MO USA; 3 Metabolomics Platform, University of Lausanne, Lausanne, Switzerland; 4 Department of Biochemistry and Molecular Biophysics, Washington University School of Medicine, St. Louis, MO USA; 5 Department of Genetics, Washington University School of Medicine, St. Louis, MO USA; 6 Howard Hughes Medical Institute, Washington University School of Medicine, St. Louis, MO USA

**Keywords:** Metabolomics, Multienzyme complexes, Metabolism, Nucleic acids

## Abstract

Rapidly proliferating cells require large amounts of nucleotides, making nucleotide metabolism a widely exploited therapeutic target against cancer, autoinflammatory disorders and viral infections. However, regulation of nucleotide metabolism remains incompletely understood. Here, we reveal regulators of de novo pyrimidine synthesis. Using uridine-sensitized CRISPR-Cas9 screening, we show that coenzyme Q (CoQ) is dispensable for pyrimidine synthesis, in the presence of the demethoxy-CoQ intermediate as alternative electron acceptor. We further report that the ADP-ribose pyrophosphatase NUDT5 directly binds PPAT, the rate-limiting enzyme in purine synthesis, which inhibits its activity and preserves the phosphoribosyl pyrophosphate (PRPP) pool. In the absence of NUDT5, hyperactive purine synthesis exhausts the PRPP pool at the expense of pyrimidine synthesis, which promotes resistance to purine and pyrimidine nucleobase analogues. Of note, the interaction between NUDT5 and PPAT is disrupted by PRPP, highlighting an intricate allosteric regulation. Overall, our findings reveal a fundamental mechanism of nucleotide balance and position NUDT5 as a regulator of nucleobase analogue metabolism.

## Abstract (web summary)

A uridine-sensitized CRISPR-Cas9 screening identifies demethoxy-CoQ as an alternative electron acceptor in the absence of CoQ, and NUDT5 as a regulator of de novo pyrimidine synthesis via its interaction with PPAT.

## Main

**Fig. 1:**
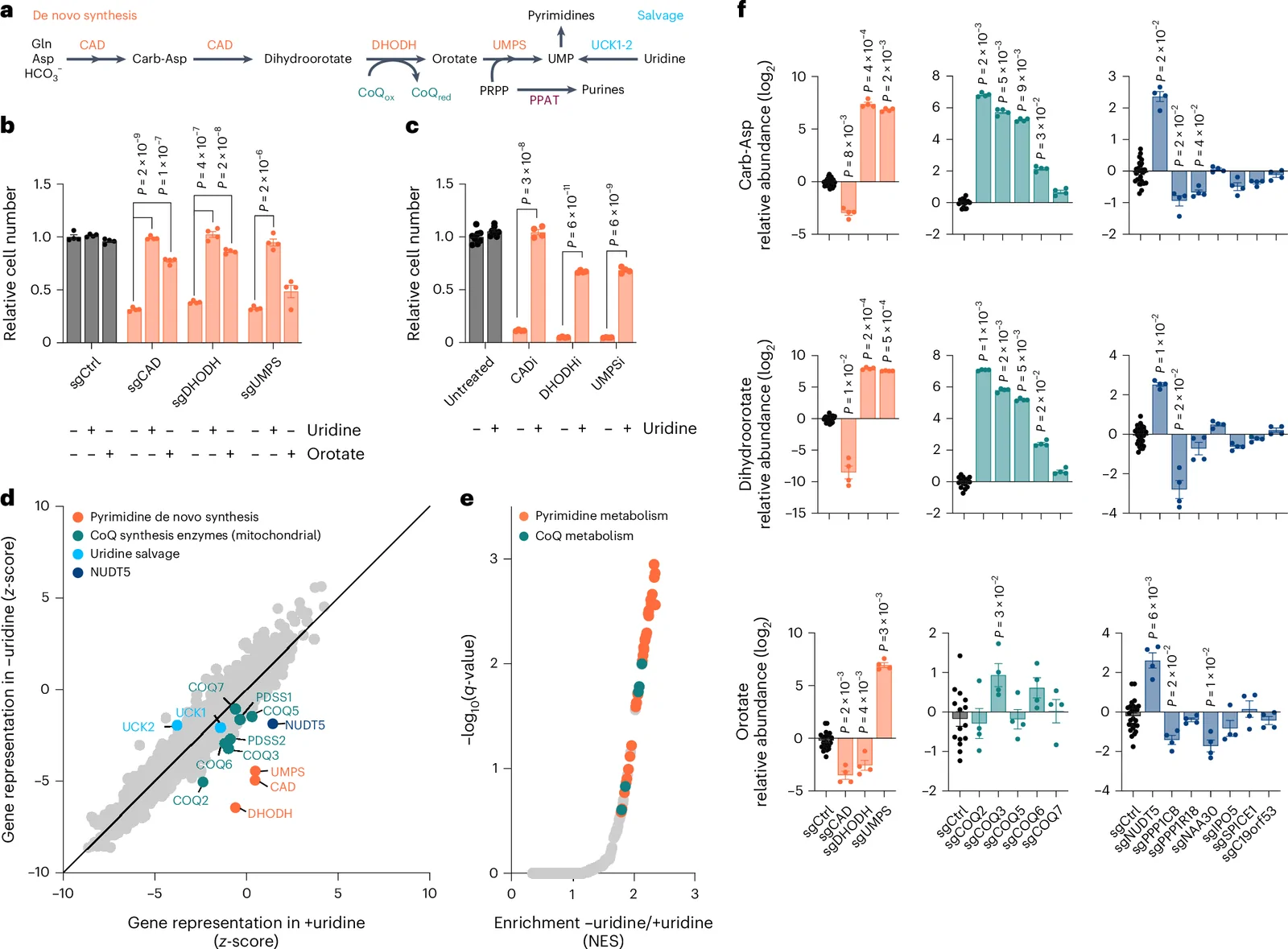
Uridine-sensitized screening identifies players in de novo pyrimidine synthesis. **a**, Simplified representation of pyrimidine de novo synthesis and salvage pathways. **b**, Proliferation assay of K562 cells with indicated knockouts over 4 days supplemented, as indicated, with 200 µM uridine or 500 µM orotate (four replicates, two-sided Student’s *t*-test with Bonferroni–Dunn correction; *P* = 2 × 10^−9^, 1 × 10^−7^, 4 × 10^−7^, 2 × 10^−8^, 2 × 10^−6^, respectively). **c**, Proliferation assay of K562 cells over 5 days supplemented, as indicated, with 200 µM uridine, 200 µM CADi (sparfosic acid), 10 µM DHODHi (brequinar) or 10 µM UMPSi (pyrazofurin) (four replicates, two-sided Student’s *t*-test with Bonferroni–Dunn correction; *P* = 3 × 10^−8^, 6 × 10^−11^, 6 × 10^−9^, respectively). **d**, Gene representation indicated by *z*-score in medium with uridine (*x* axis) or without uridine (*y* axis) from uridine-sensitized knockout screen in K562 cells. Each point is one gene. **e**, Ranked GSEA using gene ∆*Z* = *Z*_−uridine_ – *Z*_+uridine_ from uridine-sensitized screen and GO Biological Processes database with a Kolmogorov–Smirnov test. Each point represents one gene set. NES, normalized enrichment score. **f**, Relative metabolite abundances in K562 cells with indicated knockouts (four replicates, nonparametric analysis of variance (ANOVA) (Kruskall–Wallis test)). All bar graph data are mean ± s.e.m. All sgRNA-treated cell lines were analysed or further treated 8 days after sgRNA transduction. For metabolomics analysis, the medium was refreshed 4–6 h before collection. Carb-Asp, carbamoyl-aspartate; CoQ_ox_, oxidized coenzyme Q; CoQ_red_, reduced coenzyme Q; sgCtrl, control sgRNA. Source data

Pyrimidines and purines are the building blocks of life, and their cellular availability relies on two principal pathways: salvage of nucleosides and nucleobases from dietary uptake and nucleic acid turnover, and de novo synthesis from substrates such as amino acids and sugars. The latter pathway is especially crucial in rapidly proliferating cells, which must meet increased demands for nucleotides to sustain DNA replication and cell growth^1–3^. Pyrimidine de novo synthesis involves the sequential action of three key enzymes (Fig. 1a) starting with the multifunctional protein CAD (carbamoyl-phosphate synthetase II, aspartate transcarbamylase and dihydroorotase). It is followed by dihydroorotate dehydrogenase (DHODH), localized in the mitochondria, whose activity canonically relies on electron transfer to CoQ (ubiquinone), participating in the mitochondrial electron transport chain^4–6^. The final enzyme, uridine monophosphate synthase (UMPS), acts in the cytosol to link the pyrimidine ring with phosphoribosyl pyrophosphate (PRPP) to form uridine monophosphate (UMP), the precursor of all pyrimidine nucleotides. In contrast, de novo biosynthesis of purines is initiated directly on the PRPP backbone through a ten-step pathway beginning with the amidophosphoribosyltransferase PPAT (Fig. 1a). Mutations in the genes required for nucleotide biosynthesis lead to rare genetic disorders that, in the case of pyrimidine deficiency, can be treated by oral supplementation with uridine, the main substrate for pyrimidine salvage^7–9^.

Precise regulation of nucleotide biosynthesis is vital for maintaining nucleotide balance and cellular homeostasis. CAD and PPAT, as the first commitment steps of their respective pathways, are rate-limiting enzymes and are strictly regulated to control de novo nucleotide synthesis^4,10–14^. Cancer cells, characterized by rapid proliferation, rely on these pathways to maintain their enhanced metabolic needs, and both cancer and immune cells can catabolize nucleosides when glucose is scarce^15–20^, making nucleotide metabolism a prime target for therapeutic intervention. Nucleotide analogues mimic endogenous nucleotides, thereby disrupting DNA replication and RNA stability, and are widely used in therapies against cancer, autoimmunity and viral infections^1,21^. However, despite the clinical success of over 20 US Food and Drug Administration-approved analogues for treating malignancies such as leukaemia and pancreatic cancer, autoinflammatory conditions such as rheumatoid arthritis, or for blocking viral replication, resistance frequently arises due to genetic instability and competition with endogenous substrates^22–24^. These limitations underscore the need to identify new regulatory mechanisms and therapeutic targets within nucleotide metabolism to overcome resistance and improve treatment outcomes.

Here, we leverage the convergence of pyrimidine de novo synthesis and salvage pathways to design a uridine-sensitized CRISPR-Cas9 screening method to identify regulators of pyrimidine de novo synthesis. We reveal that a CoQ precursor can functionally replace CoQ for pyrimidine de novo synthesis and identify a non-catalytic role for the ADP-pyrophosphatase NUDT5 in nucleotide metabolism. We demonstrate that NUDT5 maintains PRPP levels essential for pyrimidine synthesis and mediates nucleobase analogue toxicity by inhibiting PPAT through PRPP-sensitive protein–protein interaction. Our results highlight NUDT5 as a critical and physiological node in ensuring balanced nucleotide production, thereby sensitizing cells to nucleotide analogue therapies.

## Uridine-sensitized screening identifies factors in pyrimidine synthesis

**Extended Data Fig. 1:**
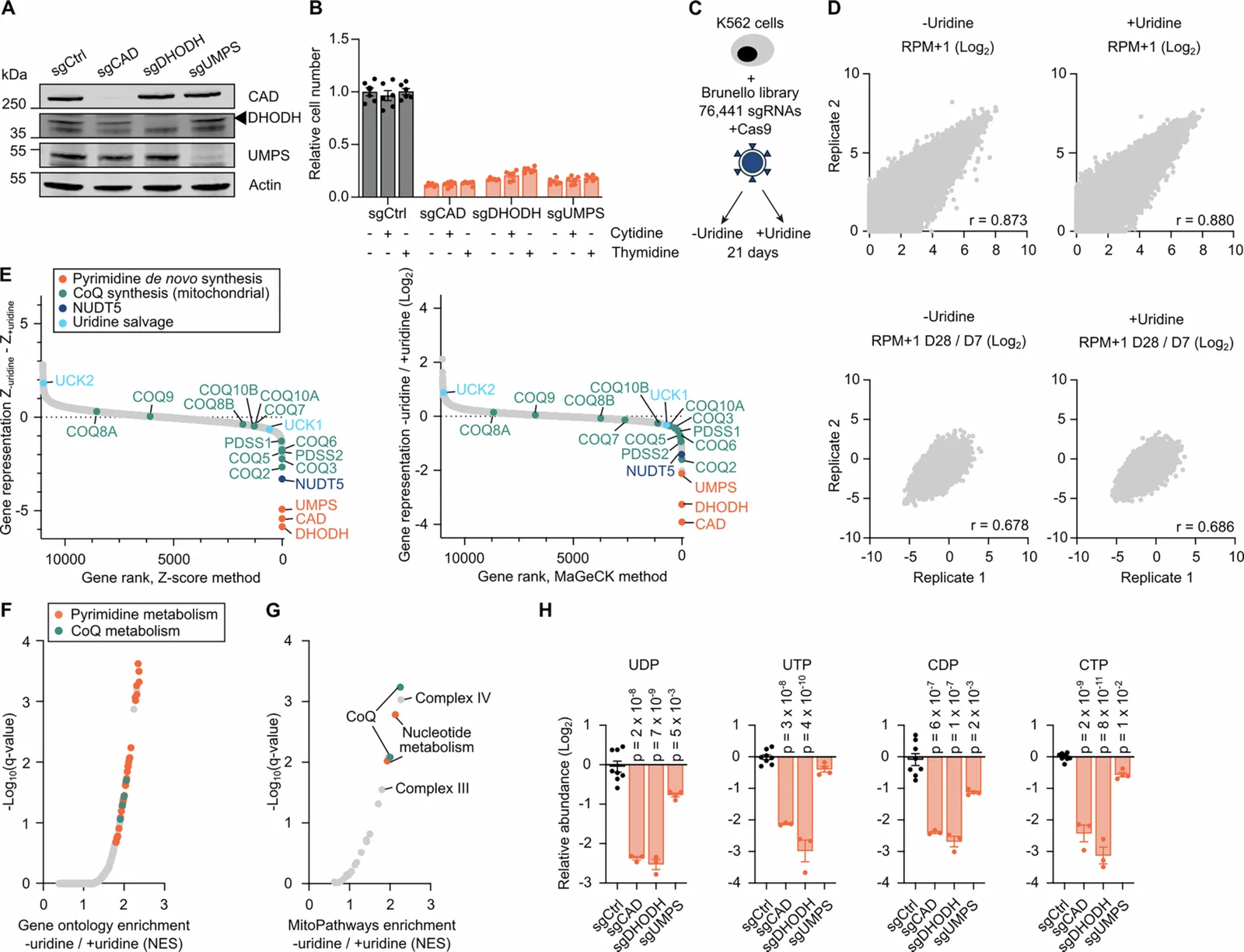
Uridine-sensitized screening discovers genes of pyrimidine synthesis. **A**. Immunoblot of K562 cells with knockout of the three key enzymes of pyrimidine de novo synthesis. **B**. Proliferation assay of K562 cells with indicated knockouts over 5 days supplemented, as indicated, with 200 µM cytidine or 25 µM thymidine (6 replicates, two-sided Student t-test with Bonferroni-Dunn correction). The lower dose of thymidine was selected due to toxicity. **C**. Representation of uridine-sensitized genome-wide knockout screen. K562 cells were infected with the Brunello genome-wide sgRNA knockout library, split into medium with or without 200 µM uridine supplementation, and cultured for 21 days. **D**. Comparison of sgRNA representation in replicate infections of CRISPR-Cas9 screening. Data are RPM + 1 or as a fold-change with day 7 post-infection (day of medium switch). Each point is one sgRNA of an expressed gene. Statistical test: Pearson’s correlation. **E**. Gene representation in medium without uridine supplementation relative to medium with 200 µM uridine, as calculated by Z-score (left) or MAGeCK (right) analysis of uridine-sensitized screen using Kolmogorov–Smirnov test. Each point is one gene. **F**. Ranked gene set enrichment analysis using gene fold-changes from MAGeCK analysis of uridine-sensitized screen and gene ontology biological processes database. Each point is one gene set. **G**. Ranked gene set enrichment analysis using gene ∆Z = Z_-uridine_ – Z_+uridine_ from uridine-sensitized screen and MitoPathways database. Each point is one gene set. **H**. Relative metabolite abundances in K562 cells with indicated knockouts (4 replicates, one-way ANOVA, p = 2 ×10^−8^, 7 x 10^−9^, 5 x 10^−3^, 3 x 10^−8^, 4 x 10^−10^, 6 x 10^−7^, 1 ×10^−7^, 2 x 10^−3^, 2 x 10^−9^, 8 x 10^−11^, 1 x 10^−2^, respectively). Bar graph data are mean ± SEM. All sgRNA-treated cell lines were analysed or further treated 8 days after sgRNA transduction. For metabolomics analysis, media were refreshed 4–6 h prior to harvesting. Abbreviations; CoQ: coenzyme Q, NES: normalized enrichment score, RPM: reads per million, sgCtrl: control sgRNA. Source data

To discover genes involved in pyrimidine metabolism, we sought to exploit the dependency on nucleoside salvage exhibited when de novo synthesis is impaired^25–27^. We used CRISPR-Cas9 to deplete the three key enzymes required for de novo pyrimidine synthesis (*CAD*, *DHODH* and *UMPS*) in K562 myelogenous leukaemia cells, which resulted in a strong reduction in proliferation (Fig. 1a,b and Extended Data Fig. 1a). We next supplemented uridine to the cell culture medium and found that it was sufficient to restore proliferation in cells genetically depleted for, or treated with inhibitors of, any of the three key enzymes (Fig. 1b,c). This contrasted with supplementation of cytidine or thymidine, two downstream pyrimidine nucleosides and with the intermediate orotate, which rescued only *CAD* and *DHODH* depletion, but not *UMPS* (Fig. 1b and Extended Data Fig. 1b).

Having confirmed dependency on uridine salvage, we next conducted a genome-wide CRISPR-Cas9 depletion screen comparing cell proliferation in the presence or absence of supplemental uridine (Extended Data Fig. 1c,d and Supplementary Table 1). We applied two analytical methods, a *z*-score-based approach^28^ and the Model-based Analysis of Genome-wide CRISPR-Cas9 Knockout (MAGeCK) algorithm^29^, both of which highlighted the three key enzymes of de novo pyrimidine biosynthesis as the top differentially essential genes in the absence of uridine, while salvage enzymes (*UCK1* and *UCK2*) were dispensable (Fig. 1d, Extended Data Fig. 1e and Supplementary Table 1). We confirmed these findings by gene set enrichment analysis (GSEA)^30,31^ using both Gene Ontology (GO; Biological Processes)^32,33^ or a curated set of mitochondrial pathways (MitoPathways)^34^ gene sets (Fig. 1e, Extended Data Fig. 1f,g and Supplementary Table 1). These analyses further identified requirement for CoQ biosynthesis and its downstream electron acceptors, complexes III and IV (CIII and CIV) of the respiratory chain, an expected result as CoQ is the canonical electron acceptor for DHODH^4–6^ and is linked to the mitochondrial respiratory chain (Fig. 1a). CoQ synthesis within the mitochondria relies on eight known catalytic enzymes, as well as accessory factors that may facilitate substrate access^35^. Among the known enzymes catalysing steps in CoQ synthesis tested in our screen, only *COQ7* did not score significantly (Fig. 1d and Supplementary Table 1). Of note, our screen also highlighted other factors not previously linked to pyrimidine biosynthesis, and by prioritizing genes with high scores using both analytical methods we selected for further investigation genes encoding the ADP-sugar pyrophosphatase (*NUDT5*), the serine/threonine-protein phosphatase PP1-b catalytic subunit (*PPP1CB*) and one of its regulatory subunits (*PPP1R18*), the catalytic subunit of N-terminal acetyltransferase C (NatC) complex (*NAA30*), an importin (*IPO5*) and two genes encoding poorly characterized proteins (*SPICE1* and *C19orf53*) (Supplementary Table 1).

Using CRISPR-Cas9, we individually depleted each of these genes, all three de novo pyrimidine synthesis enzymes, and five CoQ biosynthetic enzymes involved in CoQ head group maturation, including *COQ7*, in K562 cells. We used targeted metabolomics to analyse the levels of carbamoyl-aspartate, dihydroorotate and orotate, the three intermediates of de novo pyrimidine biosynthesis (Fig. 1a), as we reasoned that changes in their abundance would indicate the biosynthetic steps affected in these cells. In validation of this approach, we found altered levels of pyrimidine precursors in cells depleted for each of the three enzymes of de novo pyrimidine synthesis, with metabolomes characterized by (1) a profound decrease in all intermediates following *CAD* depletion; (2) accumulation of carbamoyl-aspartate and dihydroorotate, but decreased orotate following *DHODH* depletion; or (3) accumulation of all three intermediates following *UMPS* depletion; in all conditions with a significant decrease in pyrimidine nucleotides, yet with a milder effect after *UMPS* depletion, possibly illustrating lower sgRNA efficiency, compensatory pathways or traces of nucleotide precursors in the dialysed FBS (Fig. 1f and Extended Data Fig. 1h). We found that the amplitude of changes in these intermediates were several orders of magnitude larger than changes observed in proliferation rates (Fig. 1b), indicating that measurement of pyrimidine intermediates by metabolomics represents a more sensitive method for assessing defects in pyrimidine synthesis. We next analysed the metabolic profiles of our genes of interest and found that most fall into one of three major categories: *PPP1CB*, its binding partner *PPP1R18* and, to a lesser degree, *IPO5* resembled depletion of *CAD*; *COQ2*, *COQ3*, *COQ5* and *COQ6*, as well as *NAA30* resembled depletion of *DHODH*; and *NUDT5* resembled depletion of *UMPS*, illustrated by the accumulation of all three intermediates. *COQ7*, *SPICE1* and *C19orf53* showed no significant effects (Fig. 1f). Therefore, our targeted metabolomics approach validated most of the genes highlighted in our screen and assigned genes to discrete steps in de novo pyrimidine synthesis.

## Pyrimidine synthesis in the absence of CoQ

**Fig. 2:**
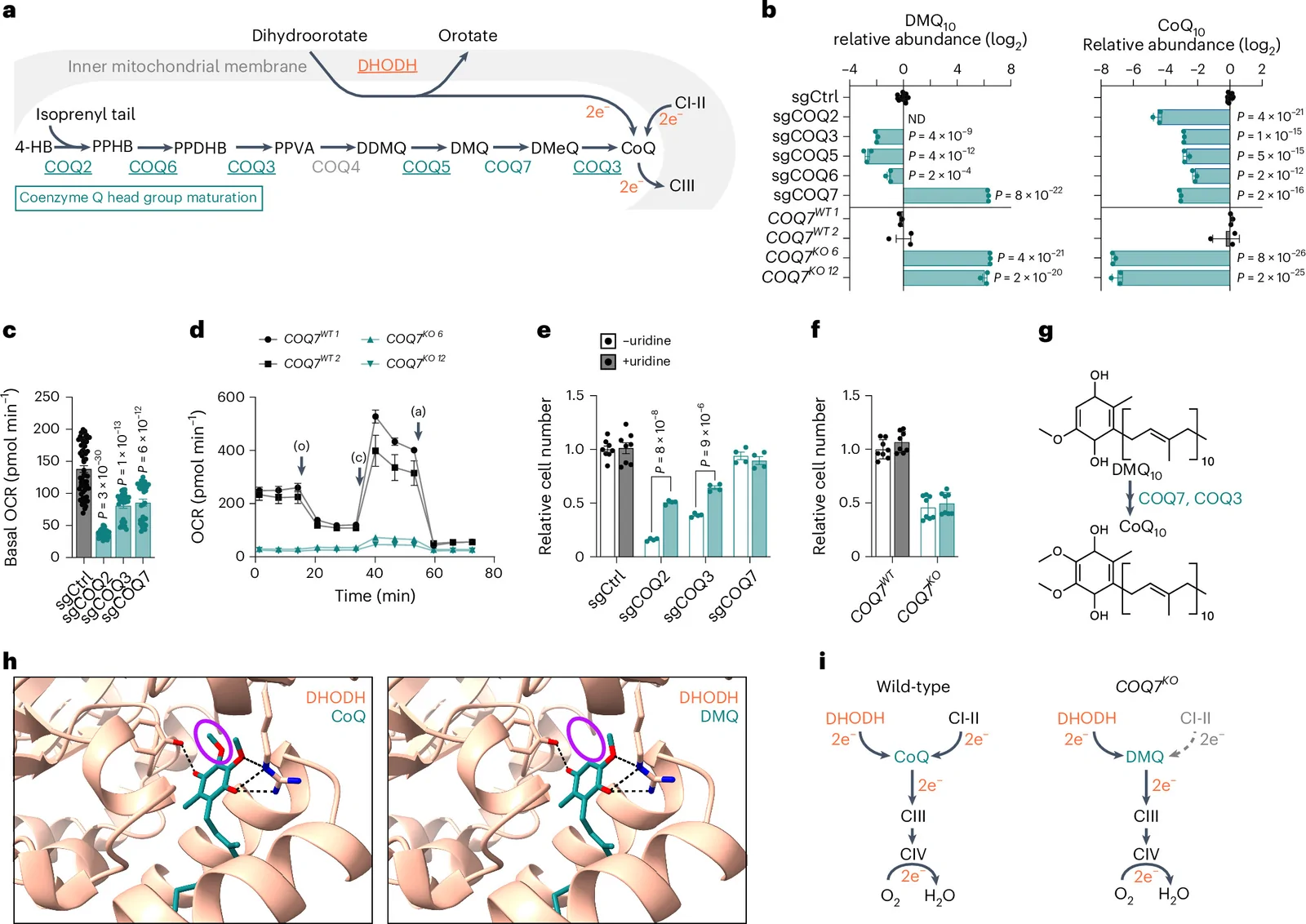
Pyrimidine synthesis, but not OXPHOS, continues in the absence of *COQ7* and mature CoQ. **a**, Simplified representation of CoQ maturation and of CoQ as the electron acceptor for DHODH or CI–II of the respiratory chain. Enzymes that were present in the Brunello library^65^ are coloured and further underlined if significant in the screen. **b**, Relative abundances of DMQ_10_ and CoQ_10_ in K562 cells with indicated KOs (three replicates, 12 for sgCtrl, *P* = 4 × 10^−9^, 4 × 10^−12^, 2 × 10^−4^, 8 × 10^−22^, 4 × 10^−21^, 1 × 10^−15^, 5 × 10^−15^, 2 × 10^−12^, 2 × 10^−16^, respectively) or in single-cell clones (four replicates, two clones each, *P* = 4 × 10^−21^, 2 × 10^−20^, 8 × 10^−26^, 2 × 10^−25^, respectively). Superscript numbers refer to clone identification. **c**, Oxygen consumption rate (OCR) of K562 cells with indicated KOs (30 replicates, 60 for sgCtrl, *P* = 3 × 10^−30^, 1 × 10^−13^, 6 × 10^−12^, respectively). **d**, OCR of *COQ7* clones (nine replicates, two clones each). (o), oligomycin; (c), CCCP; (a), antimycin A. **e**, Proliferation assay of K562 cells with indicated knockouts over 5 days supplemented, as indicated, with 200 µM uridine (four replicates, eight for sgCtrl, *P* = 8 × 10^−8^, 9 × 10^−6^, respectively). **f**, Proliferation assay of *COQ7* clones over 5 days supplemented, as indicated, with 200 µM uridine (four replicates, two clones each). **g**, Chemical structures of DMQ_10_ and CoQ_10_. **h**, AlphaFold 3 model of CoQ_10_ or DMQ_10_ binding to DHODH showing hydrogen bonds (black dashed lines). Purple oval highlights head group difference between CoQ and DMQ. **i**. Proposed model of electron transfer depending on *COQ7* expression. In wild-type conditions, CoQ is synthesized and transfers electrons for DHODH and CI–II. Without COQ7, DMQ accumulates and can sustain electron transfer for DHODH but not CI–II (grey dotted line). Data are mean ± s.e.m. Statistical tests were one-way ANOVA with Bonferroni correction for metabolomics, two-sided Student’s *t*-test with Bonferroni correction for respirometry and growth assays. All sgRNA-treated cell lines were analysed or further treated 8 days after sgRNA transduction. For metabolomics analysis, the medium was refreshed 4–6 h before collecting. 4-HB, 4-hydroxybenzoate; CoQ: coenzyme Q; DDMQ, demethoxy-demethyl-coenzyme Q; DMeQ, demethyl-coenyzme Q; DMQ, demethoxy-coenzyme Q; ND, not detected; PPDHB, polyprenyl-dihydroxybenzoate; PPHB, polyprenyl-hydroxybenzoate; PPVA, polyprenyl-vanillic acid. Source data

**Extended Data Fig. 2:**
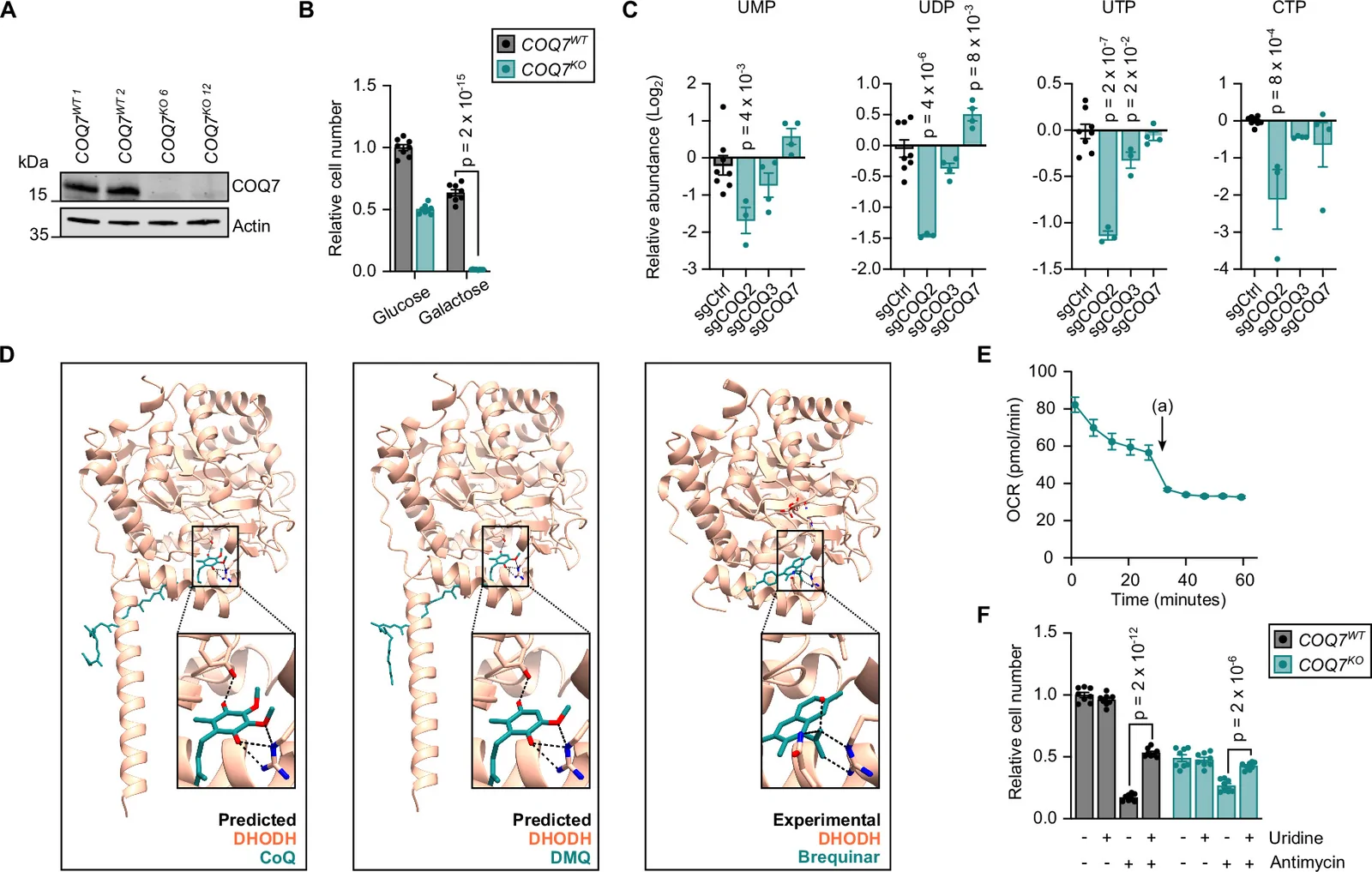
Antimycin A-dependent respiration is maintained in absence of *COQ7*. **A**. Immunoblot of *COQ7* single cell K562 clones. Superscript numbers refer to clone identification. **B**. Proliferation assay of *COQ7* clones over 6 days supplemented with 25 mM glucose or galactose (4 replicates, two clones each, p = 2 x 10^−15^). **C**. Relative metabolite abundances in K562 cells with indicated knockouts (3–4 replicates, p = 4 ×10^−3^, 4 x 10^−6^, 8 x 10^−3^, 2 ×10^−7^, 2 ×10^−2^, 8 x 10^−4^, respectively). **D**. Structures of CoQ_10_, DMQ_10_, or brequinar docking in DHODH, as determined experimentally for brequinar^39^ or using AlphaFold 3 predictive modelling for CoQ_10_ and DMQ_10_. **E**. Oxygen consumption rate (OCR) of *COQ7* clones seeded at high density (24 replicates, two clones each). (a): antimycin A. **F**. Proliferation assay of *COQ7* clones over 5 days supplemented, as indicated, with 100 nM antimycin A and 200 µM uridine (4 replicates, two clones each, p = 4 x 10^−12^, 7 x 10^−7^, respectively). Data are mean ± SEM. Statistical tests: two-sided Student t-test. All sgRNA-treated cell lines were analysed or further treated 8 days after sgRNA transduction. Abbreviations; CoQ: coenzyme Q, DMQ: demethoxy-coenzyme Q. Source data

CoQ is the canonical electron acceptor for several enzymes on the inner mitochondrial membrane, including DHODH and the respiratory chain (Fig. 2a). Thus, depletion of CoQ biosynthetic enzymes is expected to block both pyrimidine de novo synthesis and respiration^30,35,36^. Our screen effectively revealed and validated central genes in the CoQ biosynthesis pathway, with the notable exception of *COQ7* (Fig. 1f and Supplementary Table 1), which is required for conversion of the CoQ precursor demethoxy-coenzyme Q (DMQ) into demethyl-coenzyme Q (DMeQ), the final intermediate of CoQ biosynthesis^37,38^ (Fig. 2a). Given the role of CoQ in DHODH function, the observation that *COQ7* is dispensable for pyrimidine synthesis was unexpected. To investigate this anomaly, we generated K562 single-cell knockout (KO) clones (*COQ7*^*KO*^) and measured the levels of human DMQ (DMQ_10_) using lipidomics (Fig. 2b and Extended Data Fig. 2a). As expected, we found that while DMQ_10_ levels were very low in control cells and in cells depleted of four other *COQ* enzymes, it accumulated strongly in the absence of *COQ7* (Fig. 2b), showing that the conversion of DMQ_10_ could not proceed without COQ7. Similarly, we confirmed lower CoQ_10_ levels across *COQ* KOs, including *COQ7* depletion (Fig. 2b), resulting in impaired respiration and failure to thrive in galactose medium, indicating an inability to perform oxidative phosphorylation (OXPHOS) (Fig. 2c,d and Extended Data Fig. 2b). However, while uridine supplementation could partially rescue the growth defects of *COQ2*- and *COQ3*-depleted cells, as expected from the additional roles of CoQ in cellular metabolism, *COQ7* depletion had no visible effect on uridine-dependent growth and pyrimidine synthesis (Figs. 1f and 2e,f and Extended Data Fig. 2c). Thus, while mature CoQ seems strictly necessary for OXPHOS, we found through our advanced validation of the screen that pyrimidine synthesis is still possible in the absence of its electron acceptor CoQ in *COQ7*-depleted human cells, which accumulate high levels of the DMQ precursor.

To further investigate the dispensability of CoQ for pyrimidine synthesis, we used AlphaFold 3 to predict CoQ binding to DHODH. Similar to experimentally obtained structures of DHODH with the inhibitory quinone analogue brequinar^39^, our model predicted multiple interactions between CoQ and the quinone-binding pocket of DHODH, but none formed between the methoxy group in position six of CoQ, which is absent in DMQ (Fig. 2g,h and Extended Data Fig. 2d). Accordingly, DMQ docking in DHODH led to a similar model, suggesting that DHODH may accommodate both CoQ and DMQ (Fig. 2h and Extended Data Fig. 2d). Following reduction by enzymes such as DHODH, CoQ is normally oxidized by CIII and electrons are transferred through CIV to the final acceptor, oxygen. Notably, we observed residual CIII-dependent (antimycin A-sensitive) respiration in *COQ7*-depleted cells (Fig. 2d and Extended Data Fig. 2e), indicating functional reduction of oxygen despite the absence of mature CoQ. In addition, blocking CIII activity with antimycin A induced uridine auxotrophy in *COQ7*^*KO*^ cells (Extended Data Fig. 2f), showing requirement of CIII for pyrimidine synthesis even in the absence of CoQ. Together, our results indicate that CoQ is dispensible for pyrimidine synthesis and suggest that DMQ accumulation following *COQ7* depletion, while unable to support CI–II-mediated respiration, can functionally substitute CoQ to efficiently support electron transfer from DHODH to CIII, thus driving pyrimidine synthesis (Fig. 2i).

## *NUDT5* depletion causes nucleotide imbalance independently of its catalytic activity

**Fig. 3:**
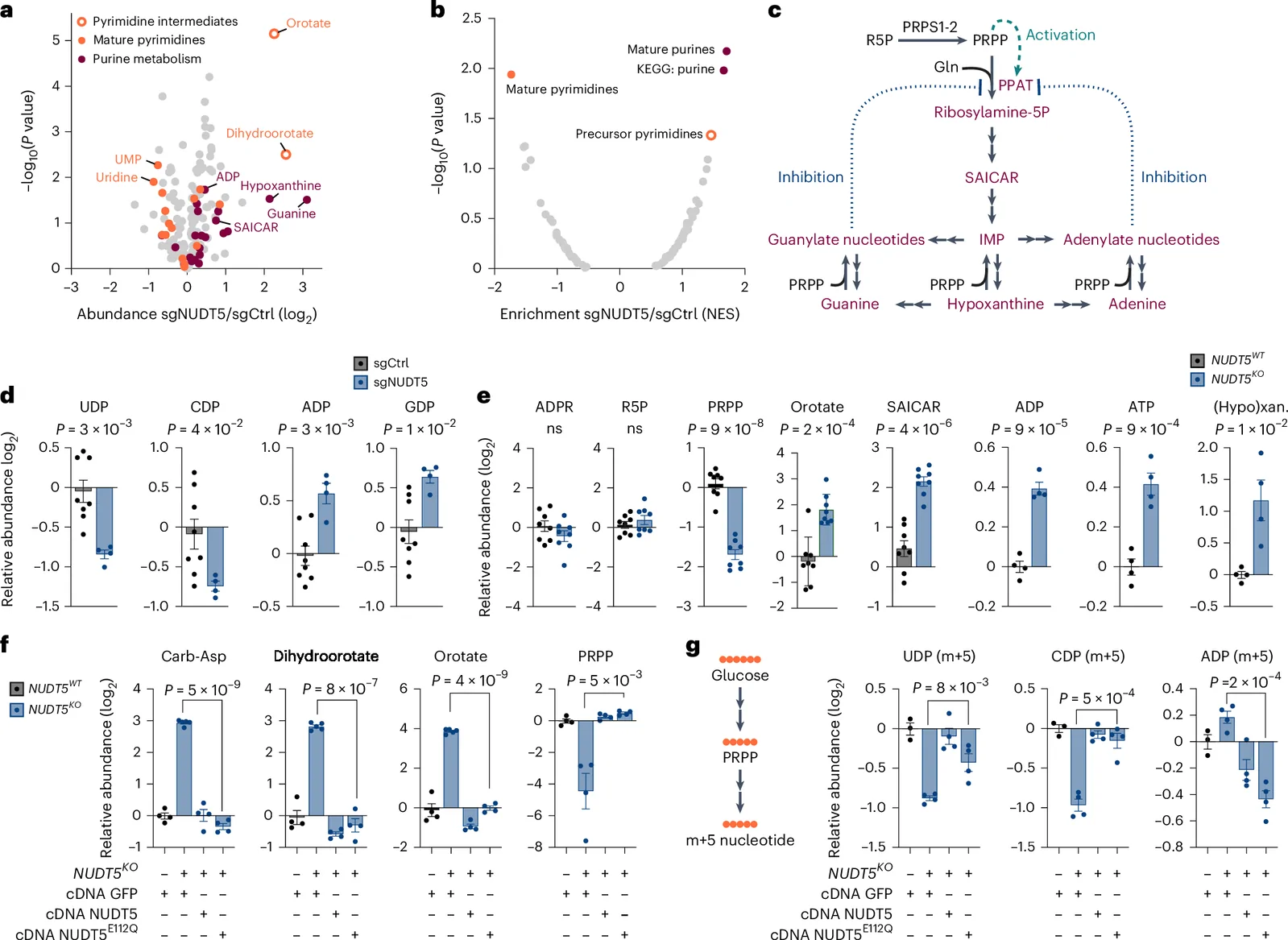
*NUDT5* depletion results in nucleotide imbalance independent of its enzymatic activity. **a**, Relative metabolite abundances comparing K562 *NUDT5* KO against control (four replicates). Each point is one metabolite. **b**, Ranked Metabolite Set Enrichment Analysis in *NUDT5* KO against control. Each point is one metabolic pathway. KEGG, Kyoto Encyclopedia of Genes and Genomes. **c**, Representation of purine biosynthesis including PRPP synthesis. PPAT is regulated by nucleotides (inhibitory feedback, blue dotted lines) and by PRPP (positive feedback, green dashed arrow). **d**, Relative metabolite abundances in K562 cells with indicated KOs (four replicates, eight for sgCtrl, *P* = 3 × 10^−3^, 4 × 10^−2^, 3 × 10^−3^, respectively). **e**, Relative metabolite abundances in *NUDT5* clones as detected using luminescence assays for ADP or ATP (three replicates, two clones each, *P* = 9 × 10^−5^, 9 × 10^−4^, respectively), fluorescence assay for (hypo)xanthine (three replicates, two clones each, *P* = 1 × 10^−2^) or targeted metabolomics for other metabolites (four replicates, two clones each, *P* = 9 × 10^−8^, 2 × 10^−4^, 4 × 10^−6^, respectively). **f**, Relative metabolite abundances in *NUDT5* clones complemented with indicated cDNAs (four replicates, *P* = 5 × 10^−9^, 8 × 10^−7^, 4 × 10^−9^, 5 × 10^−3^, respectively). NUDT5^E112Q^ is a catalytic inactive mutant of NUDT5. **g**, Representation of U-^13^C-glucose tracer with labelled carbon as orange circles (left). Relative m + 5 metabolite abundances in *NUDT5* clones complemented with indicated cDNAs and cultured for 5 h with U-^13^C-glucose tracer (four replicates) (right). Bar graph data are mean ± s.e.m. Statistical tests were a two-sided Student’s *t*-test. All sgRNA-treated cell lines were analysed or further treated 8 days after sgRNA transduction. For metabolomics analysis, the medium was refreshed 4–6 h before collecting. ADPR, ADP-ribose; (Hypo)xan., (hypo)xanthine; IMP, inosine monophosphate; R5P, ribose-5-phosphate; SAICAR, 5′-phosphoribosyl-4-(N-succinylcarboxamide)-5-aminoimidazole. Source data

**Extended Data Fig. 3:**
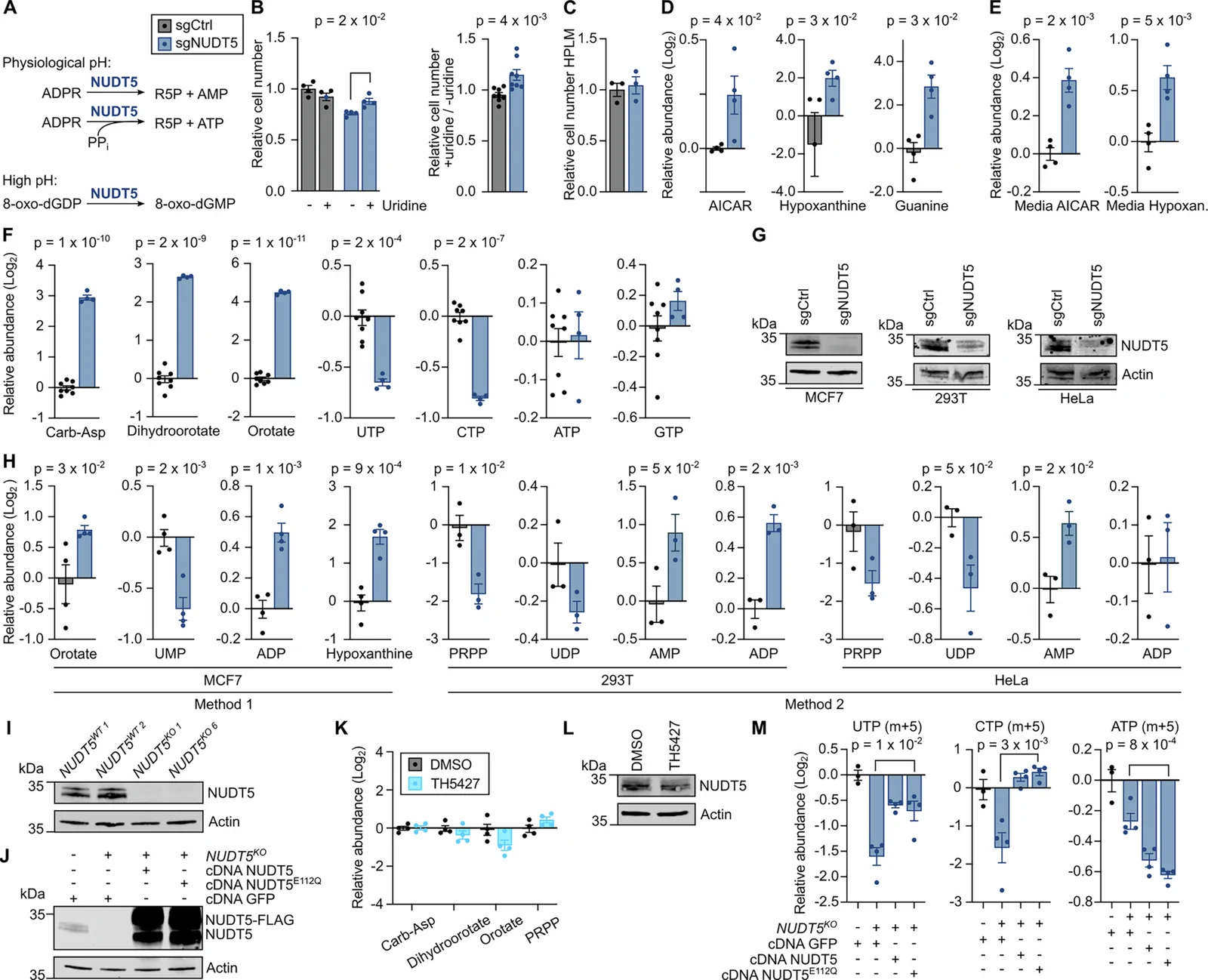
*NUDT5* depletion induces nucleotide imbalance independent of catalytic activity. **A**. Representation of reported NUDT5 enzymatic activities. **B**. Proliferation assay of K562 cells with indicated knockouts over 7 days in medium supplemented, as indicated, with 200 µM uridine and presented as a representative example (left) or fold-change (right, 8 replicates). **C**. Proliferation assay of K562 cells with indicated knockouts over 5 days in Human plasma-like medium (HPLM, 3 replicates). **D**.Relative metabolite abundances in K562 cells with indicated knockouts from multiple-pathways analysis (4 replicates). **E**. Relative metabolite abundances secreted into the medium of indicated knockouts over 6 h, normalized to medium alone (not shown). **F**. Relative metabolite abundances in in K562 cells with indicated knockouts (4 replicates, 8 for sgCtrl, p = 1 x 10^−10^, 2 x 10^−9^, 1 x 10^−11^, 2 x 10^−4^, 2 x 10^−7^, respectively). **G**. Immunoblot of *NUDT5* depletion in MCF7, 293 T, or HeLa cells. **H**. Relative metabolite abundances in MCF7, 293 T, or HeLa cells with indicated knockouts (3 replicates) and using two distinct analytical approaches for metabolite detection (Method 1: multiple-pathways targeted, Method 2: targeted). **I**. Immunoblot of *NUDT5* clones. Superscript numbers refer to clone identification. **J**. Immunoblot of *NUDT5* clones with indicated cDNA complementation. **K**. Relative metabolite abundances in K562 cells treated for 36 h with 10 µM TH5427 or DMSO (4 replicates). **L**. Immunoblot of cells in (J). **M**. Relative m + 5 metabolite abundances in *NUDT5* clones complemented with indicated cDNAs and cultured for 5 h with U-^13^C-glucose tracer (4 replicates). Bar graph data are mean ± SEM. Statistical tests: two-sided Student t-test. All sgRNA-treated cell lines were analysed or further treated 8 days after sgRNA transduction. For metabolomics analysis, media were refreshed 4–6 h prior to harvesting. Abbreviations; 8-oxo-dGDP: oxidized deoxyguanosine diphosphate, ADPR: adenosine diphosphate-ribose, AICAR: 5-phosphoribosyl-4-carbamoyl-5-aminoimidazole, Carb-Asp: carbamoyl-aspartate, PRPP: phosphoribosyl pyrophosphate, R5P: ribose-5-phosphate, SAICAR: 5’-phosphoribosyl-4-(N-succinylcarboxamide)-5-aminoimidazole, sgCtrl: control sgRNA. Source data

The top hit from our screen, apart from the three de novo pyrimidine biosynthetic enzymes, was *NUDT5* (*NUDIX5*) (Fig. 1d and Extended Data Fig. 1e), encoding for a member of the NUDIX (nucleoside diphosphate linked to moiety-X) hydrolase family. NUDT5 cleaves ADP-ribose to form ribose-5-phosphate (R5P) and AMP or ATP, depending on phosphate availability, and was also reported to cleave oxidized guanylate nucleotides at high pH^40–42^ (Extended Data Fig. 3a). We validated that uridine supplementation promotes the growth of *NUDT5*-depleted cells, including at physiological levels found in human plasma-like medium (HPLM)^43^ (Extended Data Fig. 3b,c), and, to gain a broader understanding of the influence of *NUDT5* on cell metabolism, we performed an expanded targeted metabolomics analysis on *NUDT5*-depleted K562 cells in which we observed significant accumulation of the intermediates of de novo pyrimidine synthesis and decrease of mature pyrimidines (Fig. 3a,b, Extended Data Fig. 3d and Supplementary Table 2). In contrast, we found an accumulation of both mature purines and their intermediates, validated both in cell pellets and secreted into the medium, indicating nucleotide imbalance (Fig. 3c,d and Extended Data Fig. 3d–f). We confirmed depleted pyrimidines alongside elevated purines in three additional cell lines depleted of *NUDT5* (Extended Data Fig. 3g,h), including the breast cancer line MCF7, in which earlier work on *NUDT5* was performed^41,42,44^.

To extend our investigation, we generated *NUDT5*^*KO*^ K562 single-cell clones (Extended Data Fig. 3i), in which we confirmed altered pyrimidine and purine metabolism using both targeted metabolomics and orthogonal biochemical assays (Fig. 3e). In addition, we measured ADP-ribose and R5P, two main metabolites of the NUDT5 reaction, but found no differences in their abundance, indicating that ADP-ribose catabolism from NUDT5 does not participate significantly to these pools at steady state (Fig. 3e). Having observed a similar phenotype upon either *UMPS* or *NUDT5* depletion (Fig. 1f), we next analysed the ribose donor PRPP and found that its levels were significantly decreased in *NUDT5-*depleted cells (Fig. 3e and Extended Data Fig. 3h). This observation explains the decreased pyrimidine synthesis seen upon *NUDT5* depletion, but was in part unexpected, as PRPP is also the precursor to purine synthesis, which we found elevated (Fig. 3a–c).

We next tested whether the catalytic activity of NUDT5 was required for its function in nucleotide metabolism and expressed a wild-type (WT) or a catalytically inactive mutant (E112Q)^42,45^ of NUDT5 in *NUDT5*^*KO*^ cells (Extended Data Fig. 3j). We found that both the WT or mutant NUDT5 could rescue the levels of PRPP and pyrimidine synthesis intermediates to baseline (Fig. 3f), suggesting that NUDT5 catalytic activity is not essential for its effect on pyrimidine synthesis. Similarly, a nanomolar NUDT5 catalytic inhibitor (TH5427 (ref. ^46^)) had no effect on pyrimidine intermediates nor PRPP (Extended Data Fig. 3k,l). To further validate our observations, we used uniformly labelled ^13^C-glucose, as glucose is the main precursor to PRPP (Fig. 3g). We found that the incorporation of ribose (m + 5) was attenuated in pyrimidines and elevated in adenylate purines in *NUDT5*-depleted cells (Fig. 3g and Extended Data Fig. 3m). Reintroduction of NUDT5 WT and E112Q cDNAs in these cells restored nucleotide synthesis to baseline levels (Fig. 3g and Extended Data Fig. 3m). Unlabelled (m + 0) levels of nucleotides were also affected by *NUDT5* depletion, as expected as pyrimidine synthesis is a linear pathway in which baseline and pulse-labelled nucleotide abundance depend both on the same biosynthesis and degradation rates (Supplementary Table 3). Together, our results indicate that whereas PRPP and mature pyrimidine pools are low in *NUDT5*-depleted cells, purine synthesis seems to function at a higher rate, suggesting preferential mobilization of the PRPP pool towards purines, with a detrimental effect on pyrimidine synthesis. Of note, the role of NUDT5 in maintaining nucleotide balance seems to be independent of its catalytic activity.

## NUDT5 restrains PPAT to support pyrimidine synthesis

**Fig. 4:**
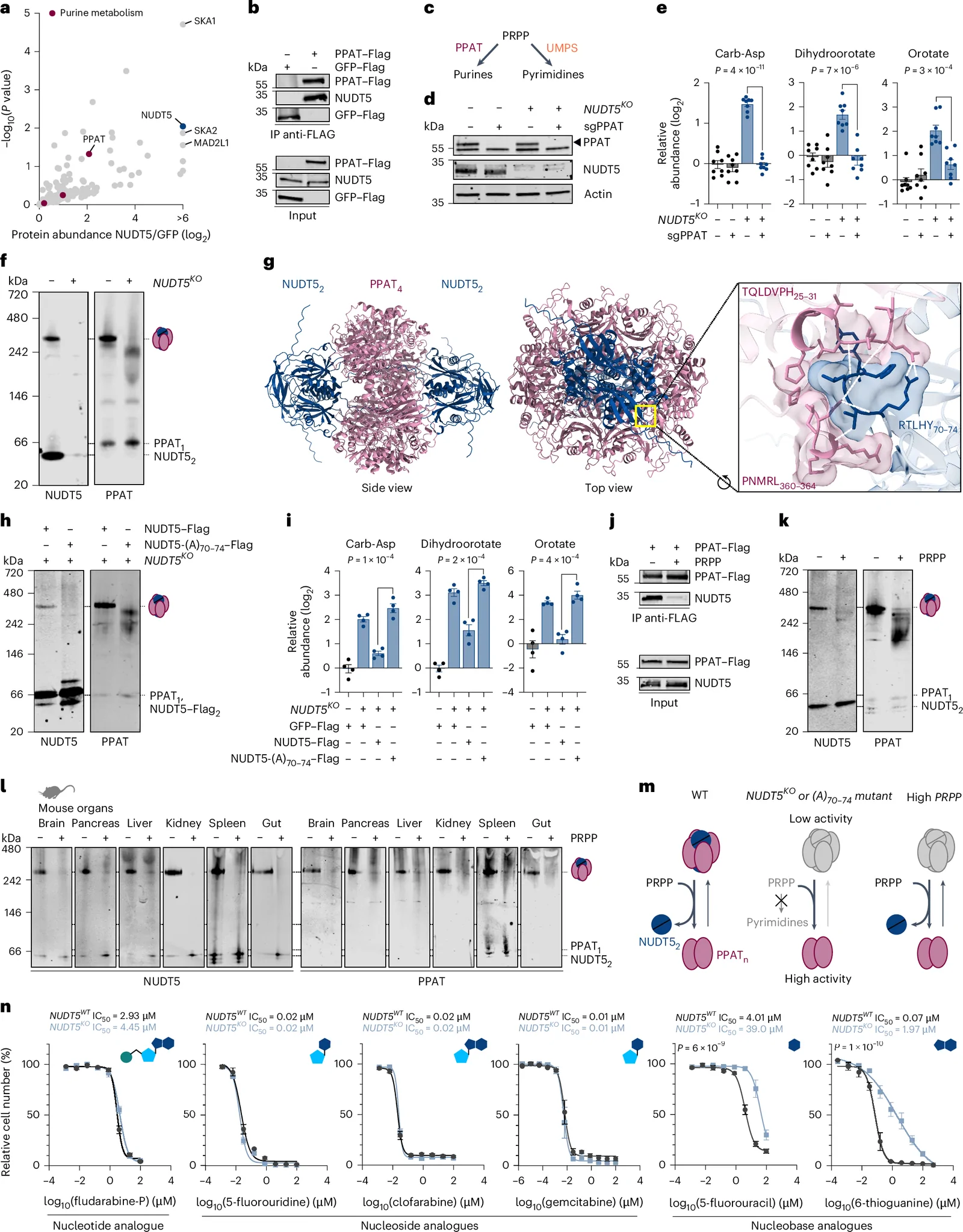
NUDT5 interacts with PPAT and prevents excessive PPAT-mediated purine synthesis at the expense of pyrimidines. **a**, Endogenous protein binding partners enriched from immunoprecipitation (IP) of Flag-tagged NUDT5 in 293T cells followed by mass spectrometry-based proteomics. Each point represents one protein. **b**, Coimmunoprecipitation from 293T cells using a Flag tag as bait. **c**, Schematic representation of PRPP use for purine or pyrimidine de novo synthesis. **d**, Immunoblot of *NUDT5* clones with indicated KOs. **e**, Relative metabolite abundances in *NUDT5* clones with indicated KOs (four replicates, two clones each, *P* = 4 × 10^−11^, 7 × 10^−6^, 3 × 10^−4^, respectively). **f**, Parallel native PAGE on *NUDT5* clones. **g**, AlphaFold 3 prediction of a complex consisting of PPAT tetramer (pink) and two NUDT5 dimers (blue), showing a zoom to the interaction interface of one NUDT5 and two PPAT molecules with amino acid residues and hydrogen bonds (dashed white lines) indicated. **h**, Parallel native PAGE on *NUDT5* clones with indicated cDNA complementation. **i**, Relative metabolite abundances in *NUDT5* clones with indicated cDNA complementation (four replicates, *P* = 1 × 10^−4^, 2 × 10^−4^, 4 × 10^−3^, respectively). **j**. Coimmunoprecipitation from 293T cells using Flag-tagged PPAT as bait with 10 mM PRPP in the IP buffer or left untreated. **k**, Parallel native PAGE on K562 cells with 10 mM PRPP in the lysis buffer or left untreated. **l**, Parallel native PAGE on indicated mouse organs with 10 mM PRPP in the lysis buffer or left untreated. **m**, Proposed model of PPAT regulation. NUDT5 binding promotes formation of the large low-activity PPAT form. When interaction with NUDT5 is disrupted, PPAT constitutively forms the small high-activity form, depleting PRPP and hindering pyrimidine synthesis. In physiological conditions, elevated PRPP similarly promotes complex dissociation and PPAT activity for purine synthesis. **n**, Proliferation assay of *NUDT5* clones over 5 days in response to a dose curve of purine and pyrimidine nucleotide analogues (two replicates, two clones each, *P* = 6 × 10^−9^, 1 × 10^−10^, respectively). A molecular representation is shown with the nitrogenous base (dark blue polygon), ribose sugar (light blue pentagon) and phosphate (green circle). Data are mean ± s.e.m. and are fitted by four-parameter logistic regression. Bar graph data are mean ± s.e.m. Statistical tests were a two-sided Student’s *t*-test. All sgRNA-treated cell lines were analysed or further treated 8 days after sgRNA transduction. For metabolomics analysis, medium was refreshed 4–6 h before collection. Source data

**Extended Data Fig. 4:**
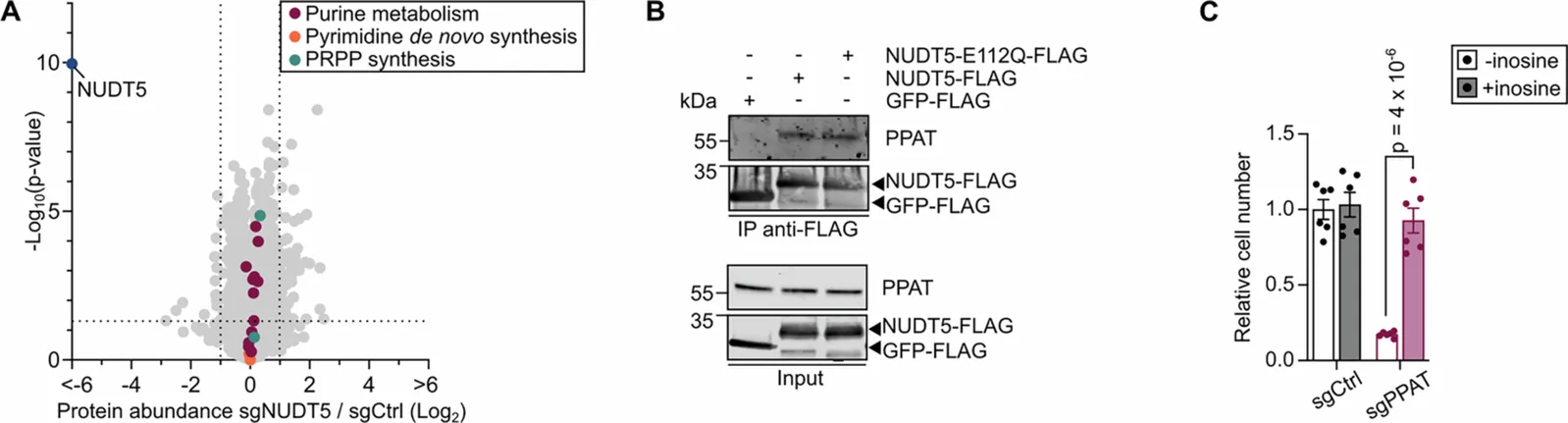
Quantitative cell proteomics of *NUDT5*-depleted cells, validation of NUDT5–PPAT interaction, and inosine supplementation of *PPAT*-depleted cells. **A**. Global proteomics comparing K562 cells with *NUDT5* knockout against control. Each point is one protein. **B**. Coimmunoprecipitation from 293 T using FLAG-tag as bait. NUDT5^E112Q^ is a catalytic inactive mutant. **C**. Proliferation assay of *NUDT5*^*WT*^ K562 clones with indicated knockouts over 5 days in medium supplemented, as indicated, with 200 µM inosine (3 replicates, two clones each, p = 4 x 10^−6^). Bar graph data are mean ± SEM. Statistical tests: two-sided Student t-test. Abbreviations; IP: immunoprecipitation, PRPP: phosphoribosyl pyrophosphate, sgCtrl: control sgRNA. Source data

Having excluded an enzymatic mechanism through which NUDT5 affects pyrimidine synthesis, we investigated changes in the proteome that occur upon *NUDT5* depletion. However, no substantial changes were observed in the abundance of enzymes involved in purine, pyrimidine or PRPP synthesis (Extended Data Fig. 4a and Supplementary Table 4). We then immunoprecipitated Flag-tagged NUDT5 and investigated its binding partners by mass spectrometry (Fig. 4a and Supplementary Table 4). We found a number of NUDT5-interacting proteins, including cytoskeletal elements, and notably PPAT, the rate-limiting and first committed enzyme required for de novo purine biosynthesis^12^ (Fig. 3c). An interaction between NUDT5 and PPAT has previously been reported in proteome-scale protein–protein interaction studies^47–49^. Because both NUDT5 and PPAT migrate at the same sizes as the immunoglobulins used for immunoprecipitation, we confirmed the interaction using either of NUDT5–Flag WT, its catalytic inactive E112Q mutant or PPAT–Flag as bait (Fig. 4b and Extended Data Fig. 4b).

PPAT and UMPS both consume and potentially compete for PRPP, a metabolite we found to be critically low in *NUDT5*-depleted cells (Fig. 4c). To determine whether PPAT may be at the origin of the pyrimidine deficiency seen in these cells, we sought to simultaneously deplete *PPAT* and *NUDT5* in an epistasis genetic experiment. However, similar to uridine dependency when pyrimidine de novo synthesis is compromised (Fig. 1b), *PPAT*-depleted cells become dependent on purine salvage, and we therefore supplemented these with inosine (Extended Data Fig. 4c). Of note, we found that *PPAT* depletion was sufficient to restore carbamoyl-aspartate, dihydroorotate and orotate to WT levels in *NUDT5*^*KO*^ cells (Fig. 4d,e). Our observations indicate a contribution for PPAT to the pyrimidine phenotype and position PPAT downstream of NUDT5.

**Extended Data Fig. 5:**
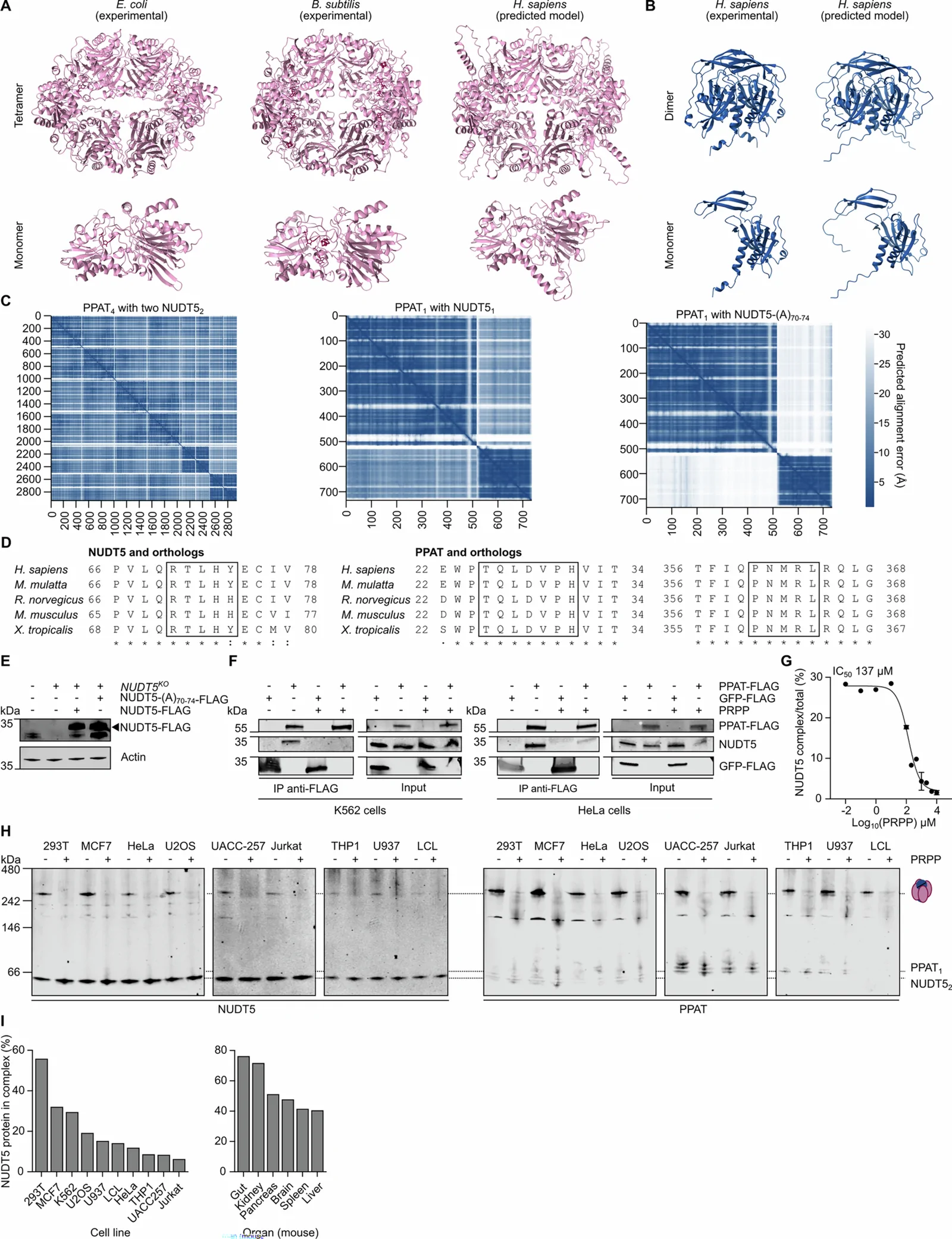
The PPAT-NUDT5 complex is conserved across species and regulated by PRPP. **A**. Structures of bacterial purF determined experimentally in complex with AMP^55,56^ and AlphaFold 3 predicted structure of human PPAT. Monomers were isolated from the tetramer structures. **B**. Structure of human NUDT5 dimer as determined experimentally^57^ or through AlphaFold 3 predictive modelling. Monomers were isolated from the dimer structures. **C**. AlphaFold 3 predicted alignment error of indicated NUDT5–PPAT complexes. **D**. Sequence alignment of NUDT5 and PPAT proteins across vertebrate species in regions predicted by AlphaFold 3 to mediate the NUDT5–PPAT interaction (black boxes). **E**. Immunoblot of *NUDT5* clones with indicated cDNA complementation. **F**. Coimmunoprecipitation from K562 or HeLa cells using FLAG-tag as bait with 10 mM PRPP in the lysis buffer or left untreated. **G**. Quantification of NUDT5 in complex with PPAT from K562 cells in response to a dose curve of PRPP in the lysis buffer, calculated from native PAGE (2 replicates). Data are mean ± SEM and are fitted by four-parameter logistic regression. **H**. Parallel native PAGE across seven cell lines with 10 mM PRPP in the lysis buffer or left untreated. **I**. Percentage NUDT5 in complex with PPAT or as a homodimer as quantified from native gels in **H** (left) and Fig. 4l (right, 1 replicate). All sgRNA-treated cell lines were analysed or further treated 8 days after sgRNA transduction. Abbreviations; IC_50_: half-maximal inhibitory concentration, IP: immunoprecipitation, PRPP: phosphoribosyl pyrophosphate, sgCtrl: control sgRNA. Source data

Seminal work from the 1970s identified the presence of PPAT in two interconvertible forms, consisting of a larger and partially inactive ~300-kDa form, and a smaller active form. We thus further investigated the NUDT5–PPAT interaction using native gel electrophoresis in K562 cells, where we predominantly observed the large ~300-kDa form with low enzymatic activity^50–52^ (Fig. 4f). Notably, this large PPAT oligomer co-migrated with NUDT5, which was also present independently as a dimer^42^, thus allowing us to confirm the interaction between the two endogenous proteins (Fig. 4f); however, when *NUDT5* was depleted, the PPAT complex dissociated (Fig. 4f), forming smaller oligomers consistent with the active form^50–52^, and suggesting the interaction is inhibitory for PPAT, as hinted to by our genetic experiment (Fig. 4e). We next aimed to model the NUDT5–PPAT complex, but no experimental structure of human PPAT currently exists, likely due to its labile iron-sulfur cluster and sensitivity to oxygen that have hindered its recombinant production^53^. We thus used AlphaFold 3 (ref. ^54^) to predict the structure of a human PPAT monomer (57 kDa) and tetramer (228 kDa), both of which exhibited strong similarities to experimentally determined bacterial PPAT homologue purF^55,56^ (Extended Data Fig. 5a). We then predicted the structure of an NUDT5 dimer, also highly consistent with experimentally determined structures^57^ (Extended Data Fig. 5b). When we added one or two NUDT5 dimers to the PPAT tetramer, we found that these can cap either side of the PPAT barrel (Fig. 4g, Extended Data Fig. 5c and Supplementary Video 1). The predicted interaction interfaces consisted of NUDT5 residues RTLHY_70–74_, unique to NUDT5 and located outside the conserved NUDIX motif (97–118), which interacts with two PPAT molecules, associated with residues TQLDVPH_25–31_ on one and PNMRL_360–364_ on the other (Fig. 4g), all of which are conserved across vertebrates (Extended Data Fig. 5d). To determine the validity of the AlphaFold 3 model, we next mutated NUDT5 residues 70–74 to alanines and reintroduced the corresponding cDNA into *NUDT5*^*KO*^ cells (Extended Data Fig. 5e). Of note, we found that NUDT5-(A)_70–74_ was unable to rescue the formation of the NUDT5–PPAT complex, consistent with the AlphaFold 3 predictions (Fig. 4h and Extended Data Fig. 5c), nor levels of pyrimidine precursors (Fig. 4i), and showing that NUDT5 must physically interact with PPAT to support pyrimidine synthesis.

We next investigated whether the NUDT5–PPAT interaction could itself be regulated. Besides its role as a substrate, PRPP is an established allosteric activator of PPAT that influences its oligomerization, as addition of PRPP to cell lysates is sufficient to convert PPAT’s large form in its active smaller form^50–52,58^. Notably, and consistent with these reports, we found that PRPP was sufficient to disrupt the interaction between NUDT5 and PPAT across immunoprecipitations in 293T, K562 or HeLa cells (Fig. 4j and Extended Data Fig. 5f). Furthermore, we observed that PRPP could induce formation of smaller PPAT oligomers in native gel electrophoresis that were similar to those seen in *NUDT5*^*KO*^ cells (Fig. 4k). Quantifying this interaction, we found that PRPP could disrupt the PPAT oligomer in K562 cells at a half-maximal concentration (IC_50_) of 0.1 mM, falling within the reported range of intracellular PRPP in pathophysiological conditions^59,60^ (Extended Data Fig. 5g). We further confirmed the existence of the NUDT5–PPAT complex across ten cell lines and six post-mitotic mouse organs, where we found on average ~33% of NUDT5 associated to PPAT, with cell type- and organ-specific variability (Fig. 4l and Extended Data Fig. 5h,i). Notably, in all cases we found that PRPP was able to disrupt the interaction of the two proteins.

Together, our genetic and biochemical observations demonstrate that NUDT5 interacts with PPAT to promote the formation of a high-molecular weight, low-activity PPAT form that can be disrupted by the allosteric activator PRPP, and that in *NUDT5*-depleted cells, constitutively active PPAT in its low-molecular weight form consumes the endogenous PRPP pool to synthesize purines, at the expense of pyrimidine synthesis (Fig. 4m).

## Loss of *NUDT5* protects against nucleobase analogue toxicity

**Extended Data Fig. 6:**
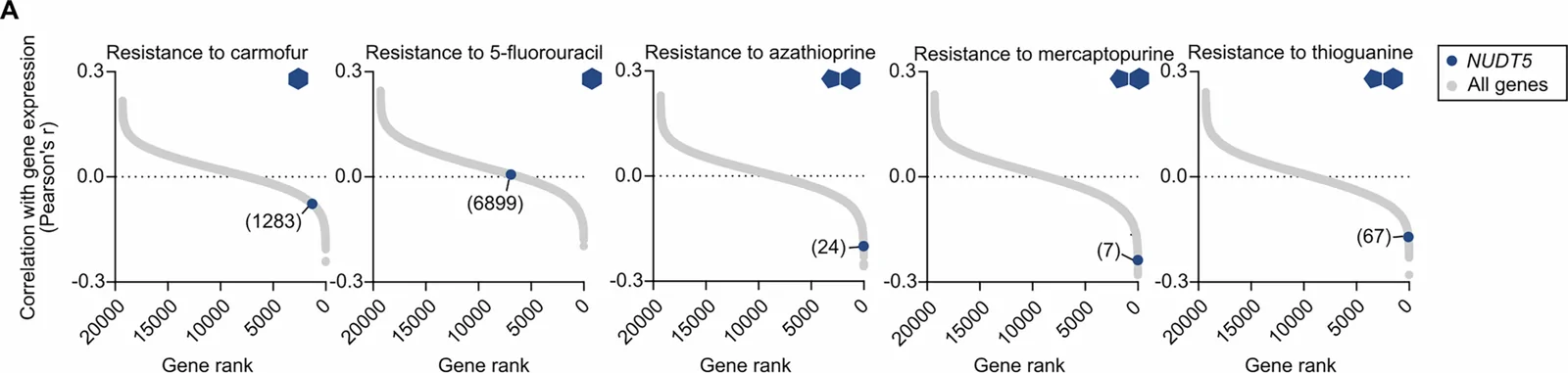
Correlation between *NUDT5* expression and sensitivity to nucleobase analogues in the Cancer Cell Line Encyclopedia. **A**. Pearson correlations of gene expression of 19,187 genes with resistance to indicated nucleobase analogues (drug perturbation scores) based on 559 cell lines. Each point is one gene and the rank of *NUDT5* is indicated. Source data

Having seen nucleotide imbalance and a strong effect on the PRPP pool in *NUDT5*^*KO*^ cells, we next tested whether *NUDT5* could affect the sensitivity of cells to nucleobase analogues, which require PRPP for their conversion into toxic nucleotide analogues^23,61–64^. This hypothesis is consistent with the results of two high-throughput screens that identified a role for *NUDT5* in 6-thioguanine (6-TG) resistance^65,66^, although in both cases the mechanism of resistance remained unclear. We first determined the IC_50_ of 5-fluorouracil (5-FU), a pyrimidine nucleobase analogue primarily metabolized by TYMP, but that can also be metabolized by UMPS^61–63,67^, and observed that *NUDT5*^*KO*^ cells were an order of magnitude more resistant to 5-FU than their corresponding WT counterparts (Fig. 4n). In contrast, sensitivity to 5-fluorouridine, the nucleosidic form of 5-FU that does not require PRPP for processing, was unchanged. We extended these observations to four other clinically used purine and pyrimidine analogues and again observed that while *NUDT5*^*KO*^ cells remained equally sensitive to all tested nucleotide and nucleoside analogues, they were consistently more resistant to nucleobase analogues that rely on PRPP, irrespective of whether the molecules were purine- or pyrimidine-based (Fig. 4n). We observed a similar trend in cells lines from the Cancer Cell Line Encyclopedia (CCLE)^68^ (Extended Data Fig. 6a). Together, our findings indicate that the role of NUDT5 in nucleotide regulation has strong implications for nucleobase analogue therapy.

## Discussion

By combining uridine sensitization and genome-wide CRISPR-Cas9 screening, we discovered factors required for de novo pyrimidine synthesis. Our approach successfully recovered *CAD, DHODH* and *UMPS* as the most important, and also highlighted a previously unidentified role for several other genes. We extensively validated such factors using metabolomics and lipidomics, assigning genes to specific steps in pyrimidine synthesis. This included all three canonical enzymes of pyrimidine synthesis, the phosphatase-related genes *PPP1CB* and *PPP1R18* whose depletion resembles that of *CAD*; genes of CoQ synthesis (*COQ2-3-5-6)* and N-terminal acetylation (*NAA30*), all of which resemble *DHODH* depletion; and the ADP-ribose pyrophosphatase *NUDT5* whose depletion resembles that of *UMPS*. We investigated NUDT5 in detail and, based on metabolic profiles, speculate that the other genes not previously linked to pyrimidine synthesis might act by regulating the activity, or the stability, of the enzymes involved in nucleotide metabolism and CoQ synthesis.

Our findings challenge the presumed indispensability of CoQ for pyrimidine synthesis. Given the relationship between DHODH and CoQ, recovery of catalytic factors of CoQ biosynthesis was an expected result, but, notably, we found that pyrimidine synthesis persists in *COQ7*-depleted cells, despite marked CoQ deficiency. Supporting this observation, the COQ7 accessory factor, COQ9 (refs. ^69,70^), did not score in our screen. While *COQ4* was not included in the Brunello CRISPR library used in this study^65^, accessory factors (*COQ8A*, *COQ8B*, *COQ10A* and *COQ10B)*, showed no uridine dependency phenotype, confirming limited or redundant functions. The dispensability of *COQ7* was unexpected and, based on the accumulation of DMQ and its molecular resemblance to CoQ (Fig. 2), we propose that DMQ may functionally substitute as an electron acceptor for DHODH. Our model is supported by the residual CIII-dependent respiration we observed upon *COQ7* depletion (Fig. 2 and Extended Data Fig. 2), as reported previously^71^, which indicates continued electron transfer in the absence of CoQ. Recent research has highlighted rhodoquinone as an alternative electron carrier able to maintain pyrimidine synthesis in the absence of CoQ^72^. Therefore, DMQ may play a similar role as it is interesting to note that CoQ, DMQ and rhodoquinone differ only by a single chemical group on the same position of the polar head group. In contrast, however, while rhodoquinone can also maintain OXPHOS^72^, we found that *COQ7* loss severely impairs respiration (Fig. 2), indicating limited ability of DMQ to function as an electron acceptor from CI–II. We propose that while DMQ can maintain pyrimidine synthesis through electron transfer to CIII and ultimately oxygen, it is insufficient to maintain electron transfers for mitochondrial CI–CII. While additional work, notably using chemically synthesized DMQ, will be required to validate electron transfer from DHODH to DMQ, our results hint at structural differences in CoQ binding sites and may pave the way for increasingly specific DHODH inhibitors.

Through genetic screening and metabolomics, we identified NUDT5 as a mediator of nucleotide balance. We validated the effect of *NUDT5* depletion on uridine-dependent growth, but found that the effect size was mild compared with the metabolic phenotype, although in both cases it reached significance (Fig. 1f and Extended Data Fig. 3b). Several technical reasons may explain this observation, including cellular adaptation, differences between pooled screen assays and individual cell lines, or traces of nucleotide precursors in dialysed FBS. It is also possible that the depletion of nucleotides might need to reach a certain threshold before presenting a strong effect on growth. Furthermore, and similar to the partial rescue of COQ2- and COQ3-depleted cells with uridine (Fig. 2e), uridine supplementation can only rescue the pyrimidine synthesis aspect of the phenotype, and in the case of NUDT5, it will not affect the potentially deleterious accumulation of purines and nucleotide imbalance, nor the other aspects of NUDT5 function that are not related to pyrimidine synthesis, such as DNA repair^42,73^, highlighting the limitations of growth assays. The difference in amplitude with the canonical enzymes of pyrimidine synthesis is coherent with our model of NUDT5 being a regulator, rather than a catalytic step, in nucleotide metabolism.

NUDT5 belongs to the NUDIX family of proteins that comprises 22 enzymes in human which are poorly conserved but share a NUDIX domain, 13 of which we detected through proteomics of K562 cells (Supplementary Table 4). This family was initially described as sanitation proteins, able to clear oxidized or otherwise modified nucleotides^74^, but more detailed characterization has revealed a broader role as pyrophosphatases affecting a range of substrates^41,75^. We now show that NUDT5 is an inhibitory binding partner to the rate-limiting purine enzyme PPAT, binds PPAT outside its conserved NUDIX domain, acts independently of its catalytic activity and that its loss promotes purine biosynthesis, depleting the endogenous PRPP pool, and thus impairing pyrimidine de novo synthesis. Of note, while PRPP also acts as an allosteric activator for PPAT^50–52,58^, our findings expand the view of PPAT regulation by identifying NUDT5 and showing that its binding is sensitive to PRPP treatment. Further research, possibly including detailed structural analysis or in vitro binding assays using purified proteins, will be needed to clarify the interplay between NUDT5- and PRPP-mediated PPAT regulation and investigate their full impact on nucleotide synthesis and macromolecular structures such as the purinosome^76^. Furthermore, nutrient availability, already known to affect PRPP levels and nucleotide synthesis^77,78^, may represent a driving factor behind this fundamental regulatory mechanism and will merit further investigation.

Our findings also position NUDT5 in the context of nucleotide analogue toxicity, including in cancer, autoinflammatory disorder, and antiviral therapies, a notion supported by other recent reports, notably those identifying germline *NUDT5* genetic variants associated with 6-TG resistance in acute lymphoblastic leukaemia^79–82^. Based on our results, two models could explain the resistance of *NUDT5*-depleted cells to nucleobase analogues: (1) the limited PRPP pool that would hinder conversion of nucleobase analogues into toxic nucleotides, and (2) competition with endogenous purines, in the case of purine analogues only. While both models are not mutually exclusive, we favour the central role of PRPP, given our experimental and CCLE data on the resistance of *NUDT5*-depleted cells not only to purine, but also to pyrimidine nucleobase analogues.

Our work highlights the power of nucleoside-sensitized genetic screens to identify genes involved in nucleotide metabolism and human disease. We expect that our approach can be extended to investigate other pathways of nucleotide metabolism, for example by screening in inosine-containing conditions (Extended Data Fig. 4c). Together, our findings are highly relevant to understanding the limitations, and improving the effectiveness, of nucleotide analogue-based therapies.

## Methods

### Animal experimentation

All animal experiments were approved by the Swiss Cantonal authorities (VD3788) and all relevant ethical regulations were followed. Animals were male C57BL/6J mice aged 12–13 weeks provided with standard laboratory chow (Safe 150) and water ad libitum, and with a standard 12-h light–dark cycle. Sex was not considered in the study design.

### Cell lines

K562 (ATCC, CCL-243), 293T (ATCC, CRL-3216), MCF7 (ATCC, HTB-22), HeLa (ATCC, CCL-2) and U2OS (ATCC, HTB-96) cells were maintained in DMEM–GlutaMAX (Gibco, 31966021) with 10% fetal bovine serum (FBS; Gibco, A5256701) and 100 U ml^−1^ penicillin–streptomycin (BioConcept, 4-01F00-H). UACC-257 (a gift from D. Fisher), Jurkat (ATCC, TIB-152), THP1 (ATCC, TIB-202), U937 (ATCC, CRL-1593.2) and LCL (Coriell, GM12878) cells were maintained in RPMI with 10% FBS (Gibco A5256701) and 100 U ml^−1^ penicillin–streptomycin (BioConcept, 4-01F00-H). UACC-257 cells were reauthenticated by STR profiling at ATCC in 2020. K562, 293T, HeLa and U2OS were acquired from ATCC less than 4 years before submission of the manuscript and were not reauthenticated. Other cell lines were not reauthenticated. All cells were cultured under 5% CO_2_ at 37 °C. Cells were periodically tested to ensure the absence of *Mycoplasma*.

### Cloning

Gene-specific single-guide RNAs (sgRNAs) were selected from the two best scoring sequences from the CRISPR-Cas9 screen and cloned into lentiCRISPR v2 vector (Addgene, 52961). The negative control (sgCtrl) was generated using guides targeting *OR2M4* and *OR11A1* that are not expressed in K562. For gene-specific complementary DNA rescue, sgRNA-resistant gene sequences were cloned into pLV-EF1a-IRES-Puro vector (Addgene, 85132). Flag-tagged green fluorescent protein (GFP) in the same vector was used as a control (Addgene, 201636). The list of sgRNA and cDNA sequences used for cloning can be found in Supplementary Table 5. Reagent requests should be addressed to the corresponding author.

### Single cell clones

Individual cloned sgRNA plasmids were electroporated into K562 cells alongside GFP using the Cell Line Nucleofector kit V (Lonza, VCA-1003) according to the manufacturer’s protocol with the T-016 program. Cells were grown for 48 h then stained with Zombie Violet (BioLegend, 423114) and fluorescence-activated single-cell sorting (FACS) was used to sort GFP^+^ Zombie^−^ cells into flat-bottom 96-well plates at one cell per well. Cells were grown for 12 days and wells with single colonies were selected for based on brightfield microscopy. Single-cell clones were expanded over 5 weeks and KOs were verified by immunoblot and by sequencing genomic DNA (gDNA), extracted using the QIAamp DNA kit (QIAGEN, 51304) according to the manufacturer’s protocol.

### Virus production for cDNA expression or gene depletion using CRISPR-Cas9 (pool format)

Lentiviruses were produced from 293T cells after transfection with a cDNA expressing plasmid, or with two plasmids containing sgRNAs to the control or target genes, as previously described^83^. Supernatant was collected 72 h following transfection, filtered through 0.45 µm and stored at −72 °C. For infections, cells at 0.5 × 10^6^ cells per ml with 10 µg ml^−1^ Polybrene (Sigma-Aldrich, TR-1003) were grown in a 1:1 ratio medium to virus supernatant for 24 h. Selection was performed with 2 µg ml^−1^ puromycin (InvivoGen, ant-pr-1) over 48 h. Cells were maintained in standard cell culture medium for 5 additional days before analysis or further experiments. Gene KO or rescue were confirmed by immunoblot and cDNA rescue with NUDT5 WT or E112Q mutant were further verified by sequencing gDNA extracted using the QIAamp DNA kit (QIAGEN, 51304), according to the manufacturer’s protocol.

### Growth assays

K562 cells were seeded at 0.05 × 10^6^ cells per ml in black flat-bottom 96-well plates (Thermo Scientific, 137101) for analysis by Prestoblue, or in flat-bottom 12-well plates for analysis by cell count. To compare glucose and galactose conditions, the test medium consisted of DMEM (Gibco, 11966-025) with 1 mM sodium pyruvate (Gibco, 11360070), 2 mM glutamine (BioConcept, 5-10K00-H), 0.2 mM uridine, 10% dialysed FBS (dFBS) (Sigma-Aldrich, F0392) and 100 U ml^−1^ penicillin–streptomycin (BioConcept, 4-01F00-H) supplemented with 25 mM glucose or galactose. The base test medium for other assays consisted of DMEM (Gibco, 31966021) with 10% dFBS (Sigma-Aldrich, F0392) and 100 U ml^−1^ penicillin–streptomycin (BioConcept, 4-01F00-H). This was supplemented as indicated in figure legends with metabolites, small molecule inhibitors or volumetric equivalents of water or dimethylsulfoxide (DMSO). Drugs purchased from MedChemExpress were sparfosic acid trisodium (HY-112732B), pyrazofurin (HY-122502), 5-fluorouridine (HY-107856), clofarabine (HY-A0005), fludarabine-phosphate (HY-B0028), and gemcitabine (HY-17026), and from Sigma-Aldrich were antimycin A (A8674), 5-FU (F6627) and 6-thioguanine (A4882). Cells were grown for 5–7 days under test conditions and proliferation was determined either using the Prestoblue dye (Invitrogen, A13262) and measuring fluorescence (ex/em 560/590 nm) with the BioTek Synergy Plate Reader (Agilent Technologies) after 1.5 h incubation at 37 °C. Background values were subtracted from Prestoblue data before analysis. For cell counts, cells were counted after 5–7 days using trypan blue-based cell counting (Vi-cell Blu counter, Beckman Coulter) and only live cells were considered.

### Denaturing polyacrylamide gel electrophoresis

Cells were collected, washed in PBS and lysed by 10 min incubation on ice in RIPA buffer (25 mM Tris, pH 7.5, 150 mM NaCl, 0.1% SDS, 0.1% sodium deoxycholate and 1% NP-40) with 1:100 protease inhibitor (Thermo Scientific, 87786) and 1:500 nuclease (Thermo Scientific, 88702). Protein concentration was quantified using DC Protein Assay kit II (Bio-Rad, 5000112). Proteins were separated by SDS–PAGE on Novex Tris-Glycine Mini Protein Gels (Invitrogen, XP08160BOX and XP10200BOX) and were transferred to nitrocellulose membranes using a wet transfer chamber with buffer consisting of 0.302% (w/v) Tris base, 1.44% (w/v) glycine and 20% ethanol in water. Transfer was verified by Ponceau S Staining Solution (Thermo Scientific, A40000278).

### Immunoblotting

Immunoblotting was performed with 5% milk or 5% bovine serum albumin (Sigma-Aldrich, A9647) in TBS (20 mM Tris-HCl, pH 7.4 and 150 mM NaCl) with 0.1% Tween-20 (TBS–Tween) and 1:500 to 1:1,000 primary antibodies or 1:5,000 secondary antibodies (LI-COR, 102673-330, 102673-328 or 102673-408). Washes were performed in TBS–Tween. Membranes were imaged by fluorescence detection at 700 and 800 nm with an Odyssey CLx Imager (LI-COR). For incubation with additional antibodies, membranes were incubated 15 min in mild stripping buffer (15% (w/v) glycine, 1% Tween-20 and 0.1% SDS, in water at pH 2.2), washed in water and reblocked. Primary antibodies were actin (Sigma-Aldrich, A3853, diluted 1:1,000), CAD (Sigma-Aldrich, HPA057266, diluted 1:500), COQ7 (Proteintech, 15083-1-AP, diluted 1:500), DHODH (Sigma-Aldrich, HPA010123, diluted 1:500), Flag M2 (Addgene, 194502-rAb, diluted 1:1,000), NUDT5 (Sigma-Aldrich, HPA019827, diluted 1:500), PPAT (Proteintech, 15401-1-AP, diluted 1:500) and UMPS (Sigma-Aldrich, HPA036178, diluted 1:500). The protein signal was quantified using Image Studio Software v.4.0 (LI-COR).

### Uridine-sensitized CRISPR-Cas9 screening

Genome-wide CRISPR-Cas9 screening was performed in K562 cells using the Brunello lentiviral library^65^ as previously described^84^. In brief, K562 cells were infected in duplicate at 500 cells per sgRNA with a multiplicity of infection of 0.3 in the presence of 10 µg ml^−1^ Polybrene (Sigma-Aldrich, TR-1003). After 24 h, cells were selected with 2 µg ml^−1^ puromycin (InvivoGen, ant-pr-1) for 48 h. On day 7 post-infection an aliquot was frozen for comparative analysis. At this time, cells were plated at 10^5^ cells per ml (equivalent to 1,000 cells per sgRNA) in DMEM–GlutaMAX (Gibco, 31966021) with 10% dFBS (Sigma-Aldrich, F0392), 100 U ml^−1^ penicillin–streptomycin (BioConcept, 4-01F00-H) and either 200 µM uridine or volumetric equivalent in sterile water. Cells were passaged every 3 days for 3 weeks and 1,000 cells per sgRNA were collected on day 28 post-infection (21 days following medium switch). Genomic DNA was extracted using NucleoSpin Blood XL kit (Machery-Nagel, 740954.20), according to the manufacturer’s protocol. Barcode sequencing, mapping and read counts were performed by the Genome Perturbation Platform (Broad Institute).

### Metabolomics analyses

#### Cell preparation

For steady-state experiments, cells were maintained in exponential phase and grown for at least 4 days in DMEM–GlutaMAX (Gibco, 31966021) with 10% dFBS (Sigma-Aldrich, F0392) and 100 U ml^−1^ penicillin–streptomycin (BioConcept, 4-01F00-H). PPAT KO cells were further supplemented with 200 µM inosine. Treatment with 10 µM TH5427 (Tocris Bioscience, 6534) or DMSO was carried out over the last 36 h. Before collection, 10^7^ cells per replicate were transferred to fresh medium for 4 h at 37 °C. Cells were collected, washed with PBS and centrifuged for 1 min at 2,000*g* at 4 °C. Supernatant was discarded and nitrogen vapour was used to displace ambient air. Pellets were flash-frozen in liquid nitrogen.

For tracer analysis, cells were first maintained in exponential phase for 7 days in DMEM–GlutaMAX (Gibco, 31966021) with 10% dFBS (Sigma-Aldrich, F0392) and 100 U ml^−1^ penicillin–streptomycin (BioConcept, 4-01F00-H). Before collection, cells were washed with PBS and seeded at 2 × 10^6^ per ml in no-glucose DMEM (Gibco, 11966-025) with 10% dFBS (Sigma-Aldrich, F0392), 100 U ml^−1^ penicillin–streptomycin (BioConcept, 4-01F00-H), 1 mM pyruvate (Gibco, 11360039) and 25 mM U-^13^C-glucose (Omicron Biochemicals, GLC-082) for 5 h at 37 °C. Cells were collected, washed with PBS and centrifuged for 1 min at 2,000*g* at 4 °C. The supernatant was discarded and nitrogen vapour was used to displace ambient air. Pellets were flash-frozen in liquid nitrogen.

#### Medium preparation

Cells were grown for 4 days in DMEM–GlutaMAX (Gibco, 31966021) with 10% dFBS (Sigma-Aldrich, F0392) and 100 U ml^−1^ penicillin–streptomycin (BioConcept, 4-01F00-H). Before collection, cells were seeded at 1 × 10^7^ per ml in fresh medium and were incubated alongside a flask of medium alone for 6 h at 37 °C. Cells were pelleted and supernatant was collected, centrifuged for 5 min at 2,000*g* at 4 °C and flash-frozen in liquid nitrogen.

#### Targeted metabolomics

Metabolites were extracted using successive freeze–thaw in methanol. Cell pellets were first resuspended in 200 µl of pre-cooled 100% (v/v) methanol containing 1 µM of ^13^C-labelled 4-hydroxybenzoic acid as an internal control and then immediately frozen in liquid nitrogen. After brief thawing, samples were centrifuged 5 min at 8,000*g* and supernatant was collected. This process was repeated twice on the remaining pellet first with 200 µl 100% (v/v) methanol and then with 100 µl 100% (v/v) water, pooling the supernatants for a final concentration of 80% (v/v) methanol. Pooled supernatants were dried under vacuum, reconstituted in 50 µl of 50% (v/v) acetonitrile:water and moved into amber glass vials for analysis.

Liquid chromatography–mass spectrometry (LC–MS) analysis was performed using a Thermo Vanquish Horizon UHPLC system coupled to a Thermo Exploris 240 Orbitrap mass spectrometer. For LC separation, a Vanquish binary pump system (Thermo Scientific) was used with a Waters Atlantis Premier BEH Z-HILIC column (100 × 2.1 mm, 1.7-µm particle size) held at 35 °C under 300 μl min^−1^ flow rate. Mobile phase A consisted of 5:95 (v/v) acetonitrile:water with 5 mM ammonium acetate (Sigma Millipore) and 200 µl l^−1^ 25% ammonium solution (Sigma Millipore). Mobile phase B consisted of 95:5 (v/v) acetonitrile:water. Descriptions of all targeted metabolomics LC methods containing times and percentages of mobile phases are given in Supplementary Table 6. Then, 1 µl of sample was injected by a Vanquish Split Sampler HT autosampler (Thermo Scientific) while the autosampler temperature was kept at 4 °C. The samples were ionized by a heated electrospray ionization (ESI) source kept at a vaporizer temperature of either 350 °C or 200 °C depending on the LC method used. Sheath gas was set to 50 units, auxiliary gas to 8 units, sweep gas to 1 unit, and the spray voltage was set to 2,500 V using negative mode. The inlet ion transfer tube temperature was kept at 325 °C with a 70% RF lens. The identity and retention time of targeted metabolites was first validated using commercial standards when available or unique MS^2^ fragments from a metabolite library (mzcloud.org) when not. Quantification of experimental samples was performed using either parallel reaction monitoring with a higher-energy collisional dissociation (30%, 40% and 50%), targeting selected ion fragments generated from the fragmentation of the hydrogen loss (H^−^) ion or targeted single-ion monitoring. A full list of the reported precursor and fragment ions is given in Supplementary Table 6. Peak integration was performed using TraceFinder v.5.1 (Thermo Scientific).

This method was used to analyse metabolites in Figs. 1, 3 and 4 and Extended Data Figs. 1, 2 and 3f,h,k,m with ‘h’ specified as ‘method 2’.

#### Targeted lipidomics

Frozen cell pellets were thawed on ice, then 150 mM KCl (50 µl) was added to each sample, followed by ice-cold methanol (600 µl) with 1 µM CoQ_8_ as internal standard (Avanti Polar Lipids). The samples were vortexed 10 min at 4 °C to lyse the cells. Ice-cold petroleum ether (400 µl) was added to extract the lipids, and the samples were vortexed again for 3 min at 4 °C. Samples were centrifuged 3 min at 1,000*g* at 21 °C and the top petroleum ether layer was collected in a new tube. The petroleum ether extraction was repeated a second time, with the petroleum ether layer from the second extraction combined with that from the first. The extracted lipids were dried under argon before being resuspended in isopropanol (40 µl) and transferred to an amber glass vial (Supelco, QSertVial, 12 × 32 mm, 0.3 ml).

LC–MS analysis was performed using a Thermo Vanquish Horizon UHPLC system coupled to a Thermo Exploris 240 Orbitrap mass spectrometer. For LC separation, a Vanquish binary pump system (Thermo Scientific) was used with a Waters Acquity CSH C18 column (100 × 2.1 mm, 1.7-µm particle size) held at 35 °C under 300 μl min^−1^ flow rate. Mobile phase A consisted of 5 mM ammonium acetate in 70:30 (v/v) acetonitrile:water with 125 μl l^−1^ acetic acid. Mobile phase B consisted of 5 mM ammonium acetate in 90:10 (v/v) isopropanol:acetonitrile with the same additive. For each sample run, mobile phase B was initially held at 2% for 2 min and then increased to 30% over 3 min. Mobile phase B was further increased to 50% over 1 min and 85% over 14 min and then raised to 99% over 1 min and held for 4 min. The column was re-equilibrated for 5 min at 2% B before the next injection. Five microliters of the sample were injected by a Vanquish Split Sampler HT autosampler (Thermo Scientific), while the autosampler temperature was kept at 4 °C. The samples were ionized by a heated ESI source kept at a vaporizer temperature of 350 °C. Sheath gas was set to 50 units, auxiliary gas to 8 units, sweep gas to 1 unit, and the spray voltage was set to 3,500 V for positive mode and 2,500 V for negative mode. The inlet ion transfer tube temperature was kept at 325 °C with a 70% RF lens. For targeted analysis, the mass spectrometer was operated in a parallel reaction-monitoring mode with polarity switching acquiring scheduled, targeted scans to CoQ_10_ H^+^ adduct (m/z 863.6912), CoQ_10_ NH4^+^ adduct (m/z 880.7177), CoQ_8_ H^+^ adduct (m/z 727.566), CoQ_8_ NH4^+^ adduct (m/z 744.5935) and CoQ intermediates: DMQ_10_ H^+^ adduct (m/z 833.6806), DMQ_10_ NH4^+^ adduct (m/z 850.7072) and PPHB_10_ H^−^ adduct (m/z 817.6504). MS acquisition parameters included a resolution of 45,000, HCD collision energy (45% for positive mode and 60% for negative mode) and 3-s dynamic exclusion. Automatic gain control targets were set to standard mode. The resulting CoQ intermediate data were processed using TraceFinder v.5.1 (Thermo Scientific). Raw intensity values were normalized to the CoQ_8_ internal standard.

#### Multiple-pathways targeted metabolomics

Cell pellets were extracted with 80% (v/v) methanol, sonicated and homogenized with ceramic beads (Precellys Cryolys). Lysates were centrifuged for 15 min at 15,000*g* at 4 °C and the supernatant was evaporated to dryness. Dried extracts were reconstituted in methanol according to total protein content as measured by a BCA assay. Samples were analysed by ultra-high performance liquid chromatography–tandem mass spectrometry (UHPLC–MS/MS), using the Triple Quadrupole mass spectrometer (6495 iFunnel, Agilent Technologies) and dynamic multiple-reaction monitoring (dMRM) acquisition mode, following previously described methods^85,86^. Two complementary liquid chromatography modes coupled to positive and negative electrospray ionization MS, respectively, were used to maximize the metabolome coverage^87^.

Raw UHPLC–MS/MS data were processed using the MassHunter Quantitative Analysis software v.B.07.00 (Agilent Technologies). Extracted ion chromatogram areas for MRM transitions were used for relative quantification. The data, consisting of peak areas of detected metabolites across all samples, were processed and filtered depending on the coefficient of variation (CV) evaluated across quality control samples that were analysed periodically throughout the batch. Peaks with analytical variability above CV of 30% were discarded.

This method was used to analyse metabolites in Fig. 3a and Extended Data Fig. 3d,e,h, with ‘h’ specified as ‘method 1’.

### Native polyacrylamide gel electrophoresis

Cells were collected, washed with PBS and lysed by 10 min incubation on ice in 1× Native Buffer (NativePAGE Sample Prep kit, Invitrogen, BN2008), 1% digitonin (Invitrogen, BN2006), 1:100 protease inhibitor (Thermo Scientific, 87786), 1:500 nuclease (Thermo Scientific, 88702) and 10 mM individual metabolites as indicated.

Frozen mouse tissue was homogenized (gentleMACS Octo Dissociator, Miltenyi Biotec) in 50 mM Tris-HCl, pH 7.4, 150 mM NaCl, 1 mM MgCl_2_, 1% NP-40, 1:100 protease inhibitor (Thermo Scientific, 87786) and 1:500 nuclease (Thermo Scientific, 88702) then centrifuged for 10 min at 3,000*g*. Supernatant was diluted in the same buffer and 10 mM metabolites were added as indicated.

Samples with metabolite supplementation were incubated 1–16 h at 4 °C with gentle agitation. Lysates were centrifuged for 10 min at 20,000 g at 4 °C and the supernatant was saved. Protein concentration was quantified using DC Protein Assay kit II (Bio-Rad, 5000112). Proteins were separated on a 4–16% native gel (Invitrogen, BN1004BOX) with 1× anode buffer (NativePAGE Running Buffer kit, Invitrogen, BN2007) and transferred to nitrocellulose membranes using a wet transfer chamber and buffer consisting of 0.302% (w/v) Tris base, 1.44% (w/v) glycine, 20% ethanol and 0.05% (w/v) SDS in water. Transfer was verified by Ponceau S Staining Solution (Thermo Scientific, A40000278). Membranes were incubated 5 min in 8% acetic acid and washed with water before proceeding to immunoblotting.

### Oxygen consumption rate

For standard respirometry, cells were seeded at 125,000 cells per well in Seahorse XF DMEM (Agilent Technologies, 103575-100) supplemented with 25 mM glucose and 2 mM glutamine, centrifuged for 1 min at 100*g* and incubated 1 h at 37 °C. The oxygen consumption rate was measured by the Agilent Seahorse XFe96 Extracellular Flux Analyzer (Agilent Technologies) using the program XF Cell MitoStress Test and successive treatment with 2 µM oligomycin A (Tocris, 4110), 1.5 µM CCCP (Sigma-Aldrich, C2759) and 1.6 µM antimycin A (Sigma-Aldrich, A8674). To determine antimycin A-dependent respiration, *COQ7* clones were instead seeded at 250,000 cells per well and tested with a single injection of antimycin at final concentration 1.6 µM. Data were analysed using Seahorse Wave Desktop Software (Agilent Technologies).

### Immunoprecipitation

The 293T cells were transfected with Flag-tagged NUDT5, NUDT5 mutants, PPAT or GFP as control using lipofectamine 2000 (Invitrogen, 11668019) according to the manufacturer’s protocol. HeLa or K562 cells were infected as described above to stably overexpress Flag-tagged PPAT or GFP. Cells were grown to 80% confluency, collected by scraping, washed with PBS and lysed by a 15–30-min incubation on ice in IP buffer (50 mM Tris-HCl, pH 7.4, 150 mM NaCl, 1 mM MgCl_2_ and 1% NP-40) with 1:100 protease inhibitor (Thermo Scientific, 87786) and 10 mM metabolites as indicated. Lysates were spun for 10 min at 20,000*g* at 4 °C and supernatants were transferred to new tubes. A 1% volume was kept aside for inputs. Anti-Flag M2 magnetic beads (Millipore, M8823) were washed in IP buffer and incubated 1–16 h with samples at 4 °C with gentle agitation. Beads were washed five times in IP buffer and changed twice to new tubes. Then, 0.1 mg ml^−1^ 3×Flag peptide (Sigma-Aldrich, F4799) in TBS was added to beads in two steps and each time incubated for 30 min at 4 °C with gentle agitation; the supernatant was saved on ice. Then, 100 µl trichloroacetic acid (Sigma-Aldrich, T9159) was added to the supernatant and incubated for 30 min at 4 °C. Samples were centrifuged for 20 min at 20,000*g* at 4 °C, the supernatant was discarded and pellets were washed in −20 °C acetone. Samples were centrifuged for 10 min at 20,000*g*, the supernatant was discarded and pellets were heated at 55 °C until dry. The pellets were resuspended in 2× SDS sample buffer for analysis by SDS–PAGE or MS.

### Proteomics analyses

#### Global proteomics

Cells were cultured 5 days in DMEM–GlutaMAX (Gibco, 31966021) with 10% dFBS (Sigma-Aldrich, F0392) and 100 U ml^−1^ penicillin–streptomycin (BioConcept, 4-01F00-H). A total of 5 × 10^6^ cells were collected and washed with PBS, and pellets were flash-frozen in liquid nitrogen. Protein extraction, library construction and analyses, and sample analyses were all carried out as described previously^88^, with minor changes. In brief, proteins were extracted using a modified iST method and dried by centrifugal evaporation. Then, a fraction of each of the eight samples were pooled for library construction and were fractionated by off-line basic reversed-phase fractionation (bRP). Dried bRP fractions were redissolved in 30 µl 2% acetonitrile with 0.5% TFA and 6 µl was injected for LC–MS/MS analysis. LC–MS/MS analyses were carried out on a TIMS-TOF Pro mass spectrometer (Bruker) interfaced through a nanospray ion source to an Ultimate 3000 RSLCnano HPLC system (Dionex) using data-dependent acquisition for library construction and data-independent acquisition for sample analysis, and analysed using Spectronaut v.17.5 (Biognosys), as previously reported^88^. In total, 99,738 precursors (7,076 protein groups) were quantified in all samples.

#### IP–MS proteomics

Samples were immunoprecipitated as described above. Digestion was carried out using the SP3 method^89^ with magnetic Sera-Mag Speedbeads (Cytiva, 45152105050250). Proteins were alkylated with 32 mM idoacetamine for 45 min at 21 °C in the dark. Beads were added at a ratio 10:1 (w/w) to samples and proteins were precipitated on beads with ethanol at a final concentration of 60%. After three washes with 80% ethanol, beads were digested in 50 µl of 100 mM ammonium bicarbonate with 1 µg trypsin (Promega V5073) and incubated 1 h at 37 °C. The same amount of trypsin was added to samples for an additional 1 h incubation. Supernatant was then recovered, transferred to new tubes, acidified with formic acid at 0.5% final concentration and dried by centrifugal evaporation. To remove traces of SDS, two sample volumes of isopropanol containing 1% TFA were added to the digests and samples were desalted on a strong cation exchange plate (Oasis MCX, Waters) by centrifugation. Digests were washed with 1% TFA in isopropanol then 0.1% formic acid with 2% acetonitrile. Peptides were eluted in 200 µl of 80% acetonitrile, 19% water and 1% (v/v) ammonia, and dried by centrifugal evaporation. Samples were then analysed using LC–MS/MS analyses were carried out on a TIMS-TOF Pro mass spectrometer (Bruker) interfaced through a nanospray ion source to an EvoSep One liquid chromatography system (EvoSep), as described previously^90^.

### ADP/ATP assay

Cells were collected, washed in PBS and seeded at 10^6^ cells per ml in white flat-bottom 96-well plates (Thermo Scientific, 136101). Cellular ADP and ATP levels were measured using the ADP/ATP Ratio Assay kit (Sigma-Aldrich, MAK135) according to the manufacturer’s protocol with a BioTek Plate Reader (Agilent Technologies).

### (Hypo)xanthine assay

Cells were collected, washed with PBS and lysed by a 10-min incubation on ice in Assay Buffer (Abcam, ab155900) at 2 × 10^7^ cells per ml. (Hypo)xanthine concentration was measured using the fluorometric protocol of the Xanthine/Hypoxanthine Assay kit (Abcam, ab155900) in black flat-bottom 96-well plates (Thermo Scientific, 137101). Fluorescence was measured at ex/em 535/587 nm with the BioTek Synergy Plate Reader (Agilent Technologies).

### Data analysis

#### CRISPR screen data analysis

For analysis by *z*-score, sgRNA read count data were processed using an approach previously described^28^. In brief, data were normalized to reads per million and transformed to log_2_ space. A log_2_ fold change for each sgRNA in each medium condition was determined relative to the mean day 7 control read count (before medium switch). The mean log_2_ fold change was calculated across sgRNAs for each gene and results were averaged across two infection replicates. Low-expression genes (transcripts per million (TPM) < 1 in DepMap 24Q2 dataset^68^) were used to define the mean and s.d. of a null distribution for each medium condition and *z*-scores for each gene were defined based on this distribution. These low-expression genes and their corresponding sgRNA were excluded from further analyses. sgRNA read count data from day 28 (day 21 post-infection) were used as input for MAGeCK (v.0.5.9.2)^29^ with default parameters and using the medium condition supplemented with uridine as the reference.

#### Gene set enrichment analysis

GSEA was performed using GSEA^30,31^ v.4.2.2 on genes ranked according to ∆*Z* = *Z*_−uridine_ – *Z*_+uridine_ or by gene log_2_ fold change as calculated by MAGeCK. Enrichment was performed against the GO Biological Processes database c5.go.bp.v2023.2.Hs.symbols or against the MitoPathways database^34^ without collapse. For analysis using the GO database, maximum size exclusion was set to 50 and minimum to 2. For analysis using the MitoPathways database, minimum size exclusion was set to 15. All other parameters were kept as default. The top 50 negatively enriched pathways were manually annotated for their relationship to either pyrimidine biosynthesis or CoQ metabolism.

#### Metabolite set enrichment analysis

Metabolites were mapped to their KEGG^91^ IDs using MetaboAnalyst v.6.0 ‘Compound ID Conversion’ tool (https://www.metaboanalyst.ca/). Metabolites that were not successfully found were mapped manually where possible and were otherwise excluded. KEGG pathway identifiers for metabolism pathways were taken from their website (https://www.genome.jp/kegg/pathway.html on 24 October 2024), excluding the subsections ‘Xenobiotics degradation and metabolism’ and ‘Chemical structure transformation maps’. The list of metabolites for each pathway was obtained using the KEGGREST v.1.44.1 package (https://bioconductor.org/packages/KEGGREST) in R v.4.4.1. These data were used to generate a metabolite database and four additional pathways were added manually, delimiting mature purines or pyrimidines from the intermediate metabolites of their respective de novo synthesis pathways. Metabolite set enrichment analysis was performed using GSEA^30,31^ v.4.2.2 on metabolites ranked according to their log_2_ fold change (sgNUDT5/sgCtrl) in abundance. Enrichment was performed against the custom database described above without collapse. Maximum size exclusion was set to 500 and minimum to 3, all other parameters were kept as default.

#### Global proteomics

Analyses were conducted with the Perseus software package (v.1.6.15.0)^92^. Standard protein contaminants were removed according to an established analytical pipeline (available at the Protein Analysis Facility and using the software package above), data were log_2_-transformed and only proteins quantified in at least four samples of one group were kept. Missing values were imputed based on normal distribution using Perseus default parameters. Student’s *t*-tests were carried out and the log_2_ fold change over control samples (sgCtrl) was calculated.

#### IP–MS proteomics

Analyses were carried out using an in-house developed software tool (available on https://github.com/UNIL-PAF/taram-backend). Standard protein contaminants were removed according to an established analytical pipeline (available at the Protein Analysis Facility and on GitHub, detailed above), data were log2-transformed and only proteins quantified in at least two samples of one group were kept, resulting in 2,949 protein groups. Missing values were imputed based on a normal distribution with a width of 0.3 s.d., downshifted by 1.8 s.d. relative to the mean. Student’s *t*-tests were carried out and the log_2_ fold change over control samples (GFP–3×Flag) was calculated. Data were filtered to exclude proteins that had not been reliably detected in both NUDT5–Flag replicate samples. Remaining data were queried against the Contaminant Repository for Affinity Purification (CRAPome) database *H.* *sapiens* Single Step Epitope tag AP-MS^93^, and proteins with an average spectral score >2.5 were excluded as common IP–MS contaminants.

#### AlphaFold 3 predictive modelling

Protein structures and interactions were modelled using AlphaFold Server^54^, and the highest confidence prediction was selected. AlphaFold 3 was used to model docking of metabolites to protein complexes. ChimeraX v.1.10 was used to view predicted models and to calculate hydrogen bonds.

#### Cancer Cell Line Encyclopedia correlation analysis

Gene expression (OmicsExpressionProteinCodingGenesTPMLogp1.csv, v.24Q4) and drug treatment (Repurposing_Public_24Q2_Extended_Primary_Data_Matrix.csv) datasets were obtained from the CCLE Dependency Map portal^94^. For each of the analysed drugs, drug perturbation scores were correlated with gene expression scores across cell lines. Pearson’s correlation coefficients were calculated using R v.4.5.0. For thioguanine, drugs named ‘thioguanine’ and ‘tioguanine’ were combined by taking the mean of their drug perturbation scores for each cell line. All 559 cell lines with gene expression scores and drug perturbation scores for the analysed drugs were included.

#### Statistics and reproducibility

Statistical analyses as described in the figure legends were performed using Prism v.10 (GraphPad Software) and exact *P* values are shown where *P* < 0.05. All data are expressed as mean ± s.e.m. and all Student’s *t*-tests were two-sided. No statistical methods were used to predetermine sample sizes but our sample sizes are similar to those reported in previous publications^15,88,90^. Samples were not randomized and not randomly assigned to experimental groups. Data distribution was assumed to be normal but this was not formally tested. Data collection and analysis were not performed blind to the conditions of the experiments, as data blinding is generally not considered relevant to cell culture experiments. Representative immunoblots and native gels are representative of a minimum of three independent replicates. Validation of coimmunoprecipitation in other cell lines was performed once. No data were excluded.

### Reporting summary

Further information on research design is available in the Nature Portfolio Reporting Summary linked to this article.

## Supplementary information

Reporting Summary

Supplementary Table 1 Uridine-sensitized CRISPR-Cas9 screen. **a**, sgRNA read count data from expressed genes. For each sgRNA, read counts and reads per million are indicated for two replicate infections on day 7 (day of medium switch) or day 28 post-infection in medium supplemented with 200 µM uridine (plus-u condition) or water (minus-u condition). **b**, *z*-score analysis of screen. **c**, MAGeCK analysis of screen using robust rank aggregation algorithm. **d**, Required gene sets from gene set enrichment analysis ranked by ∆Z = Z_-uridine_ – Z_+uridine_ and using GO biological processes gene sets. **e**, Required gene sets from GSEA ranked by MAGeCK log_2_ fold change and using Gene Ontology biological processes gene sets. **f**, Required gene sets from GSEA ranked by ∆Z = Z_-uridine_ – Z_+uridine_ and using MitoPathways gene sets. GSEA data are analysed using a Kolmogorov–Smirnov test.

Supplementary Table 2 Multiple-pathways metabolomics. **a**, Abundance of metabolites detected through multiple-pathways targeted metabolomics on K562 cells depleted of *NUDT5* or control and associated statistics (two-sided Student’s *t*-test with false discovery rate correction for multiple testing). **b**, Metabolite set enrichment analysis of above. Pathway names follow KEGG nomenclature, except where added manually to distinguish nucleotide de novo synthesis. For metabolomics analysis, the medium was refreshed 4–6 h before collection.

Supplementary Table 3 Tracer metabolomics. **a**, Abundance of (m + 0) and (m + 5) labelled metabolites in *NUDT5* clones complemented with indicated cDNAs and cultured for 5 h with U-^13^C-glucose tracer.

Supplementary Table 4 Proteomics data. **a**, Label-free quantification of proteins detected through global proteomics on K562 cells depleted of *NUDT5* or control, including statistics calculated using imputed values. **b**, Label-free quantification of proteins detected through IP–MS proteomics of Flag-tagged NUDT5 or GFP control, including statistics calculated using imputed values and average spectral counts (SCs) from the CRAPome database. Missing values are shown as ‘NaN’.

Supplementary Table 5 sgRNA and cDNA sequences used in this study for individual gene depletion or expression.

Supplementary Table 6 **a**, Mass table for targeted metabolomics analysis. **b**, Timing details of liquid chromatography methods for targeted metabolomics analysis.

Supplementary Video 1 Interaction between PPAT and NUDT5. AlphaFold 3 molecular modelling of PPAT tetrameric complex and association with two dimers of NUDT5.

## Source data

Source Data Fig. 1 Statistical source data.

Source Data Fig. 2 Statistical source data.

Source Data Fig. 3 Statistical source data.

Source Data Fig. 4 Statistical source data.

Source Data Fig. 4 Unprocessed gels.

Source Data Extended Data Fig. 1 Statistical source data.

Source Data Extended Data Fig. 1 Unprocessed gels.

Source Data Extended Data Fig. 2 Statistical source data.

Source Data Extended Data Fig. 2 Unprocessed gels.

Source Data Extended Data Fig. 3 Statistical source data.

Source Data Extended Data Fig. 3 Unprocessed gels.

Source Data Extended Data Fig. 4 Statistical source data.

Source Data Extended Data Fig. 4 Unprocessed gels.

Source Data Extended Data Fig. 5 Statistical source data.

Source Data Extended Data Fig. 5 Unprocessed gels.

Source Data Extended Data Fig. 6 Statistical source data.

## Data Availability

All raw MS proteomics data together with raw output tables are available via the Proteomexchange data repository (www.proteomexchange.org) with accession IDs PXD060320 and PXD060353. Source data are provided with this paper, and include statistical data and unmodified images of all immunoblots.
