# Supplementary figures and images for: Uridine-sensitized screening identifies demethoxy-coenzyme Q and NUDT5 as regulators of nucleotide synthesis
Source: Nat Metab. 2025 Nov 13;7(11):2221–35. doi: 10.1038/s42255-025-01419-2 (PMC12638251; doi:10.1038/s42255-025-01419-2)

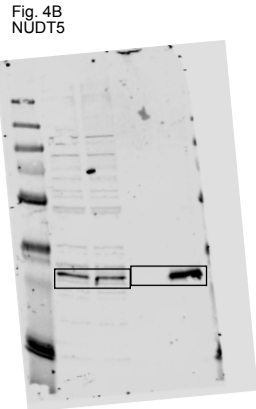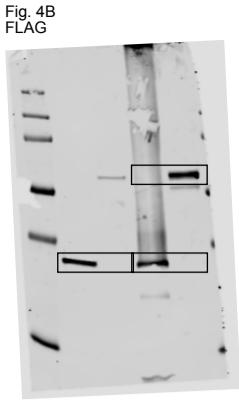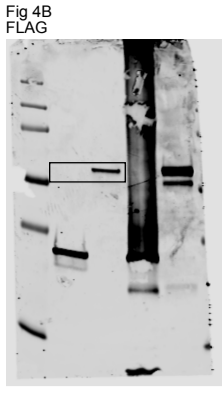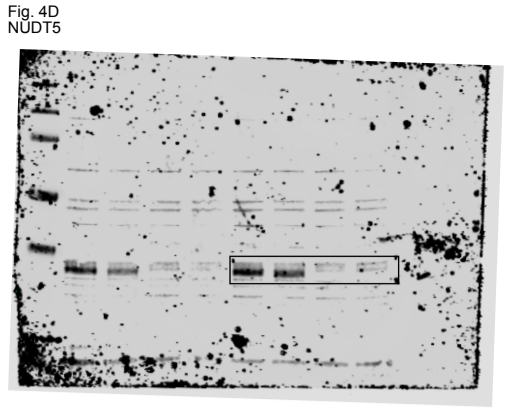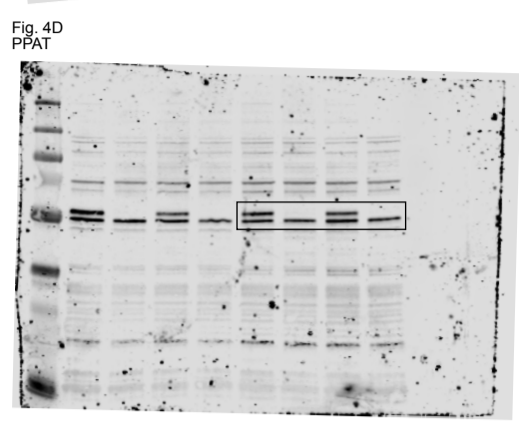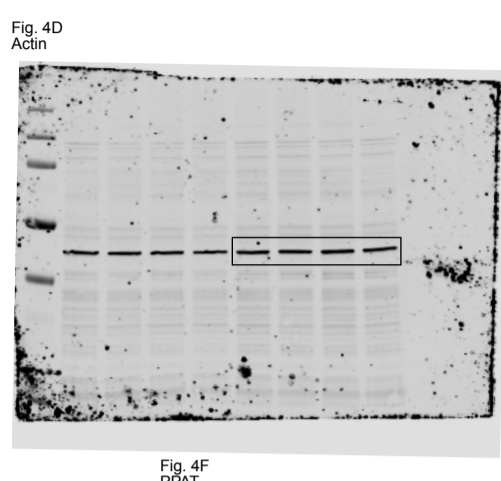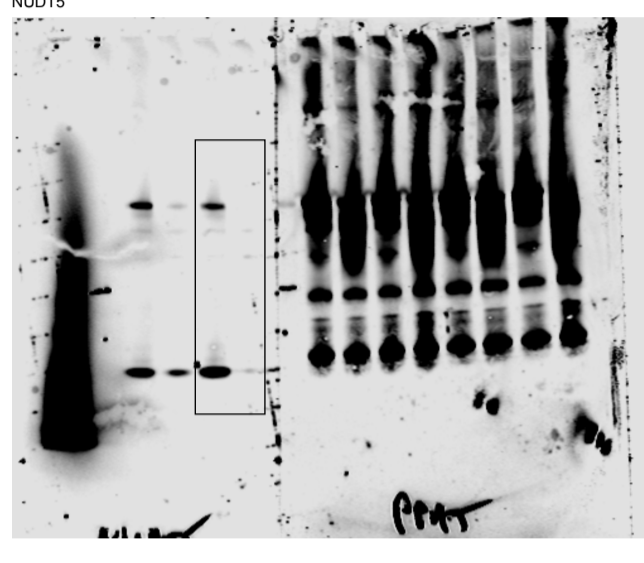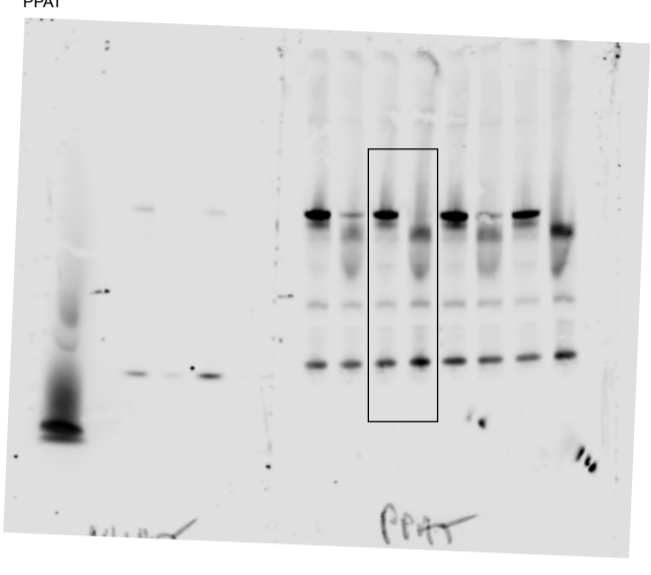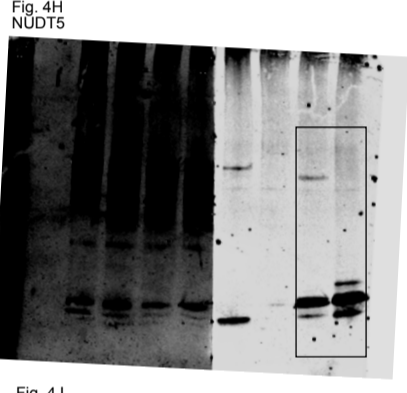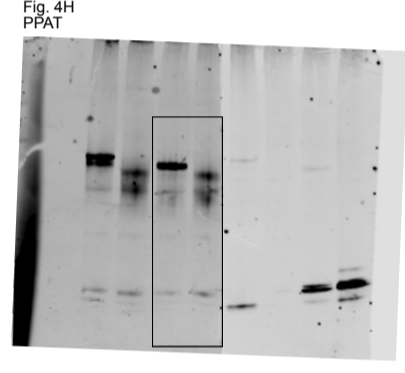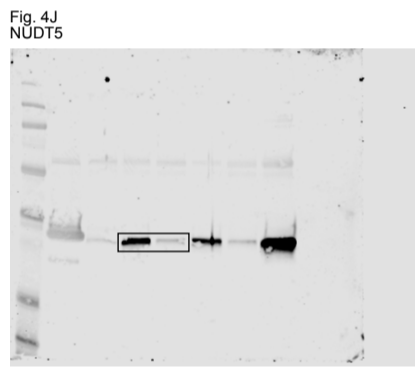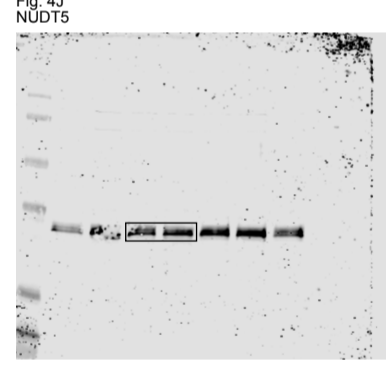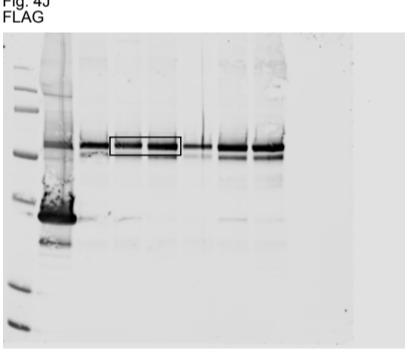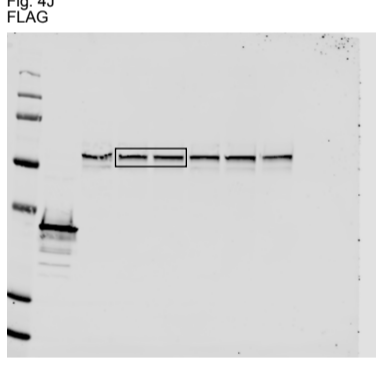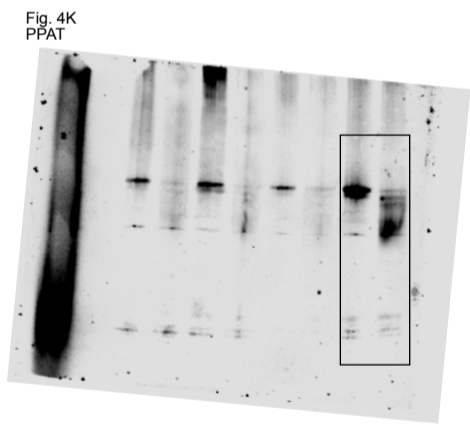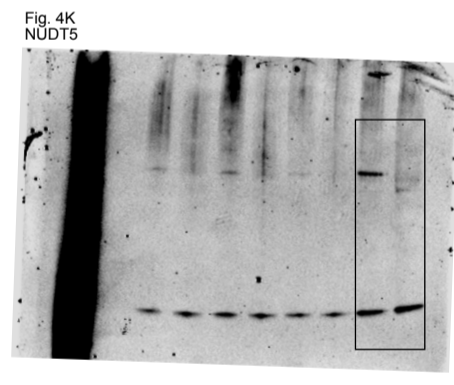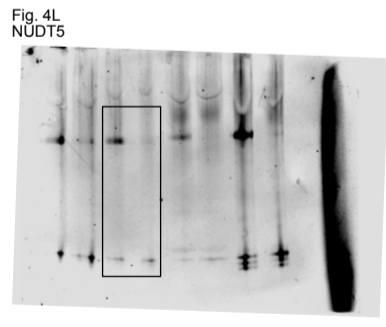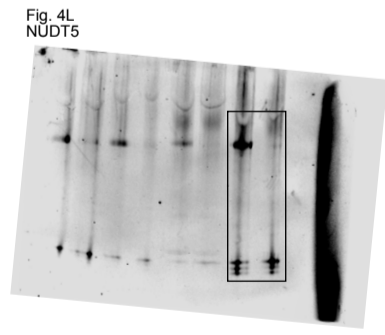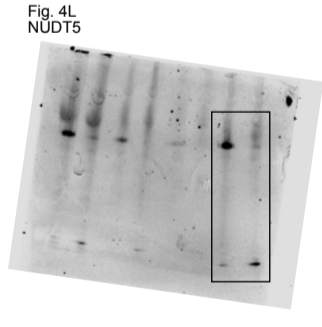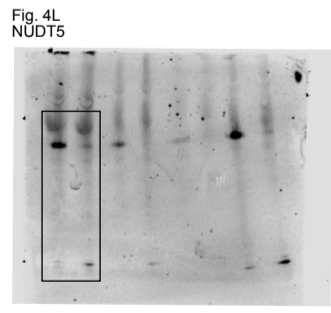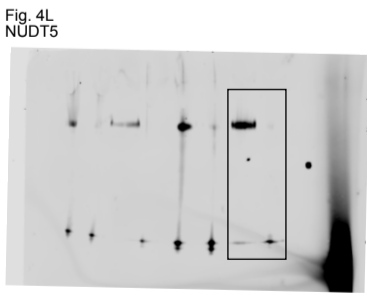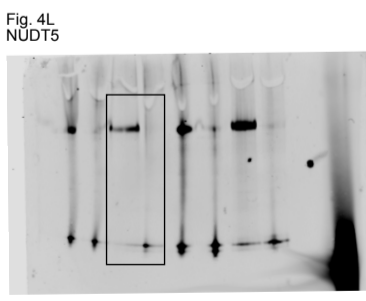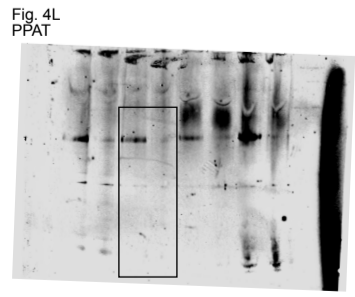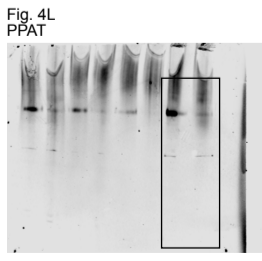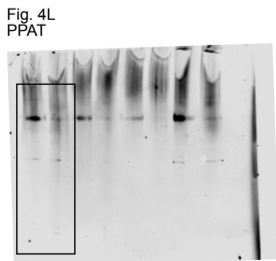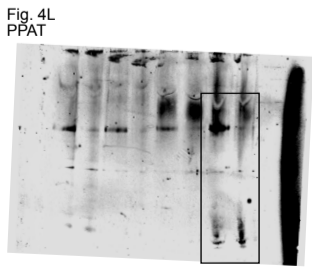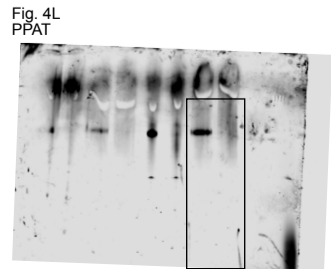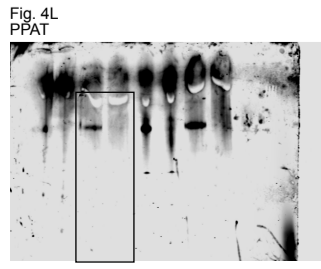

Supplement: Supplementary file 13 — Unprocessed gels. [file 42255_2025_1419_MOESM13_ESM.pdf]

Unmodified immunoblots

Associated with Extended Data Figure 2

ED Fig. 2A

Actin

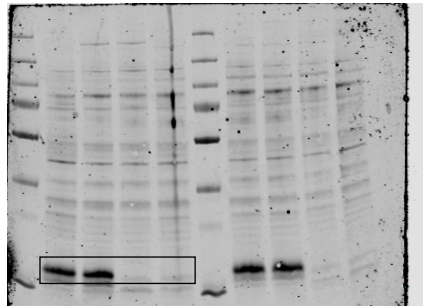

ED Fig. 2A

COQ7

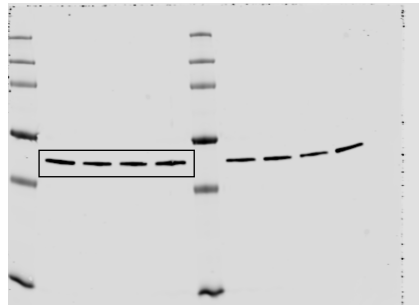

Supplement: Supplementary file 17 — Unprocessed gels. [file 42255_2025_1419_MOESM17_ESM.pdf]
