# Supplementary material for: Uridine-sensitized screening identifies demethoxy-coenzyme Q and NUDT5 as regulators of nucleotide synthesis
Source: Nat Metab. 2025 Nov 13;7(11):2221–35. doi: 10.1038/s42255-025-01419-2 (PMC12638251; doi:10.1038/s42255-025-01419-2)

Unmodified immunoblots

Associated with Extended Data Figure 1

ED Fig. 1A
CAD

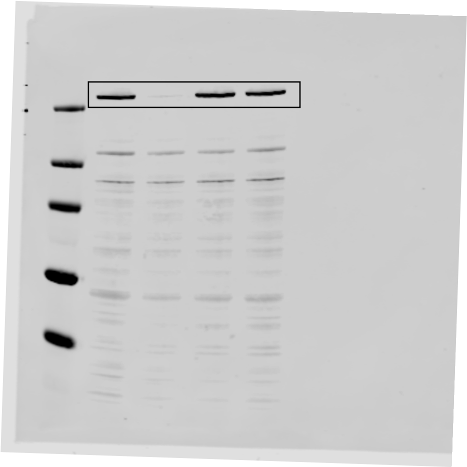

ED Fig. 1A
DHODH

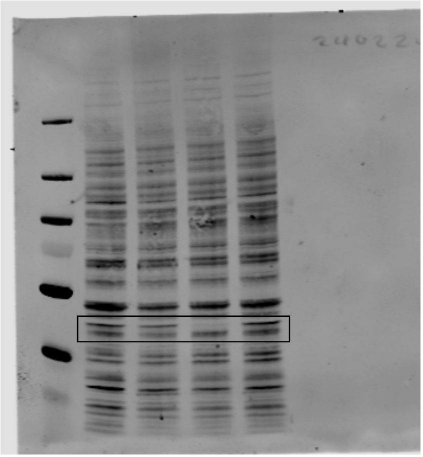

ED FIg. 1A
UMPS

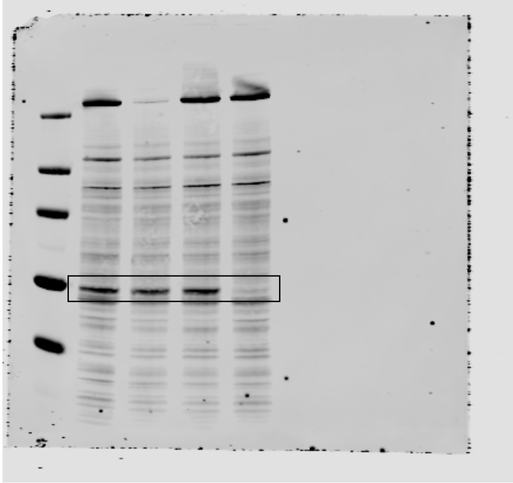

ED Fig. 1A
Actin

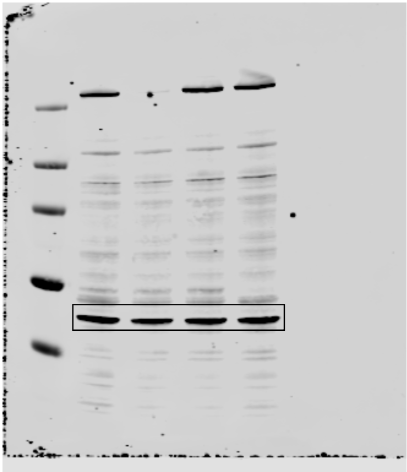

Supplement: Supplementary file 15 — Unprocessed gels. [file 42255_2025_1419_MOESM15_ESM.pdf]
