# Supplementary material for: Uridine-sensitized screening identifies demethoxy-coenzyme Q and NUDT5 as regulators of nucleotide synthesis
Source: Nat Metab. 2025 Nov 13;7(11):2221–35. doi: 10.1038/s42255-025-01419-2 (PMC12638251; doi:10.1038/s42255-025-01419-2)

Unmodified immunoblots
Associated with Extended Data Figure 5

ED Fig. 5E
NUDT5

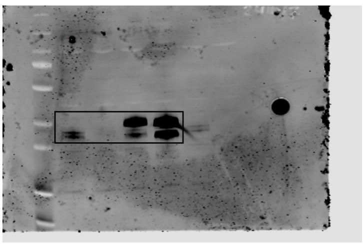

ED Fig. 5E
Actin

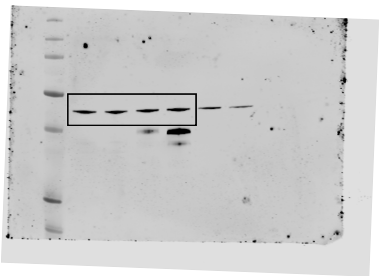

ED Fig. 5F
PPAT

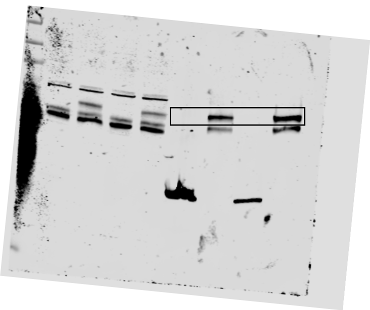

ED Fig. 5F
NUDT5

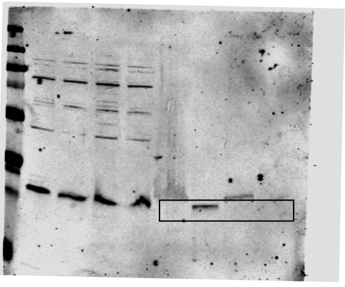

ED Fig. 5F
FLAG

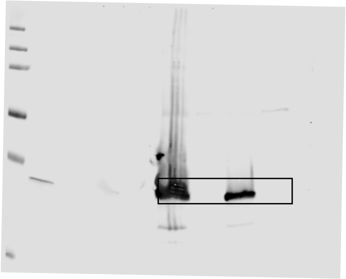

ED Fig. 5F
PPAT

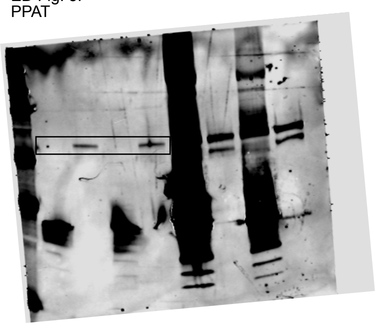

ED Fig. 5F
NUDT5

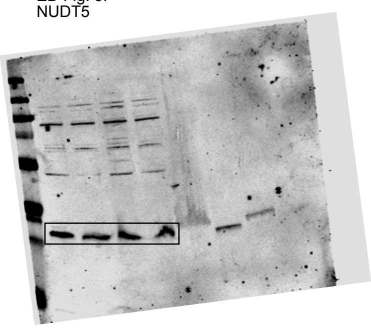

ED Fig. 5F
FLAG

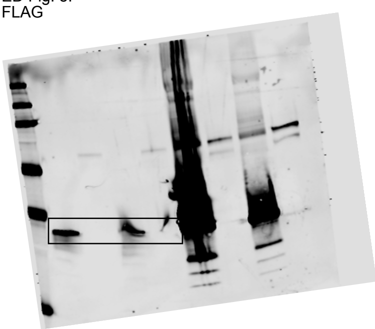

ED Fig. F5
PPAT

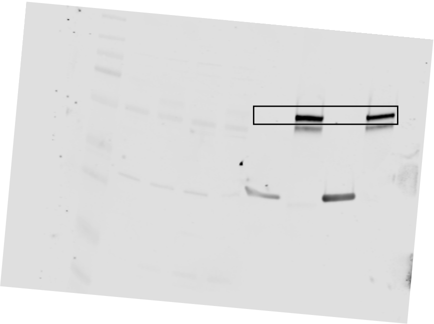

ED Fig. 5F
NUDT5

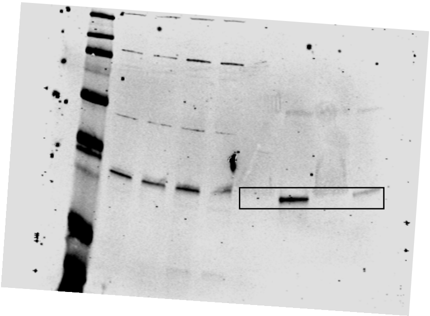

ED Fig. 5F
FLAG

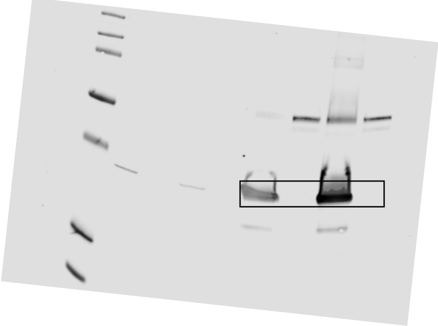

ED Fig. 5F
PPAT

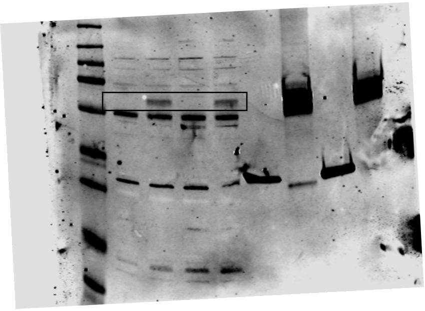

ED Fig. 5F
NUDT5

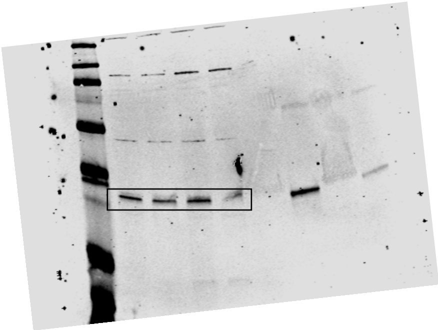

ED Fig. 5F
FLAG

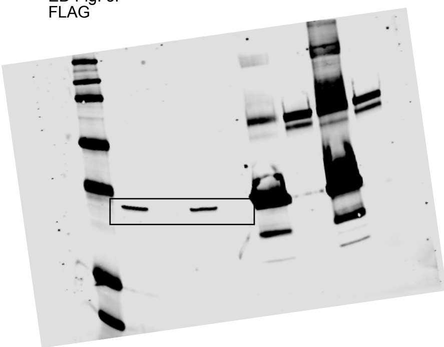

ED Fig. 5H
NUDT5

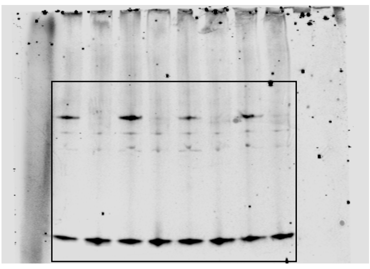

ED Fig. 5H
NUDT5

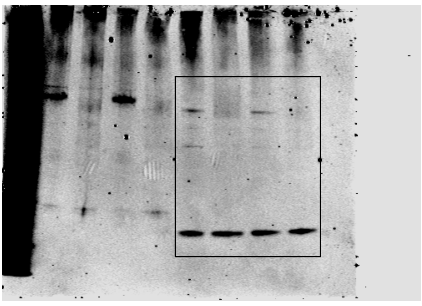

ED Fig. 5H
NUDT5

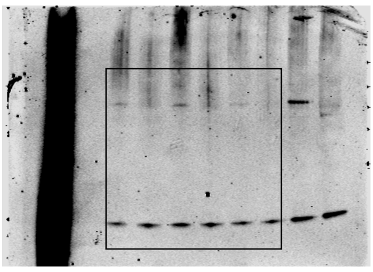

ED Fig. 5H
PPAT

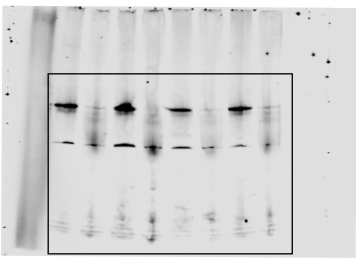

ED Fig. 5H
PPAT

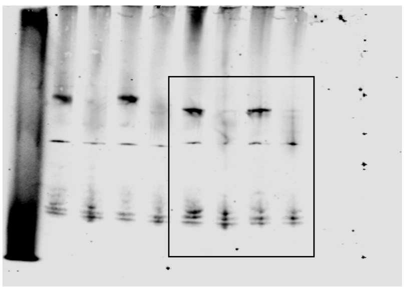

ED Fig. 5H
PPAT

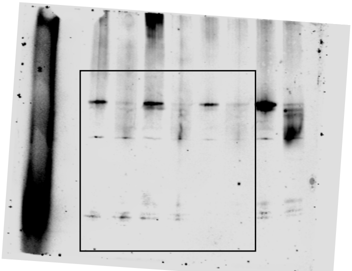

Supplement: Supplementary file 23 — Unprocessed gels. [file 42255_2025_1419_MOESM23_ESM.pdf]
